# Molecular Modeling of Viral Type I Fusion Proteins: Inhibitors of Influenza Virus Hemagglutinin and the Spike Protein of Coronavirus

**DOI:** 10.3390/v15040902

**Published:** 2023-03-31

**Authors:** Sophia S. Borisevich, Vladimir V. Zarubaev, Dmitriy N. Shcherbakov, Olga I. Yarovaya, Nariman F. Salakhutdinov

**Affiliations:** 1 Laboratory of Chemical Physics, Ufa Institute of Chemistry Ufa Federal Research Center, 450078 Ufa, Russia; 2 Laboratory of Experimental Virology, Saint-Petersburg Pasteur Institute, 197101 Saint Petersburg, Russia; zarubaev@pasteurorg.ru; 3 State Research Center of Virology and Biotechnology VECTOR, Rospotrebnadzor, 630559 Koltsovo, Russia; dnshcherbakov@gmail.com; 4 Department of Medicinal Chemistry, N.N. Vorozhtsov Novosibirsk Institute of Organic Chemistry SB RAS, 630090 Novosibirsk, Russia; anvar@nioch.nsc.ru

**Keywords:** viral fusion proteins type I, influenza virus, hemagglutinin, coronavirus, S-protein, SARS-CoV-2, molecular modeling, molecular docking, molecular dynamic simulations

## Abstract

The fusion of viral and cell membranes is one of the basic processes in the life cycles of viruses. A number of enveloped viruses confer fusion of the viral envelope and the cell membrane using surface viral fusion proteins. Their conformational rearrangements lead to the unification of lipid bilayers of cell membranes and viral envelopes and the formation of fusion pores through which the viral genome enters the cytoplasm of the cell. A deep understanding of all the stages of conformational transitions preceding the fusion of viral and cell membranes is necessary for the development of specific inhibitors of viral reproduction. This review systematizes knowledge about the results of molecular modeling aimed at finding and explaining the mechanisms of antiviral activity of entry inhibitors. The first section of this review describes types of viral fusion proteins and is followed by a comparison of the structural features of class I fusion proteins, namely influenza virus hemagglutinin and the S-protein of the human coronavirus.

## 1. Surface Viral Proteins

An important step in the life cycle of an enveloped virus in the process of penetration and infection of a cell is the fusion of the viral membrane with the membrane of the target cell [1,2,3,4]. All enveloped viruses, including deadly human pathogens such as the human immunodeficiency virus (HIV), Ebola virus, or severe acute respiratory syndrome coronavirus 2 (SARS-CoV-2) fuse the envelope of the virus and cell membrane by fusion proteins [4,5]. The main purpose of the fusion proteins of these viruses is to bind to the receptor and mediate subsequent conformational rearrangements, which finally lead to the unification of lipid bilayers and the formation of a fusion pore through which the viral genome enters the cytoplasm of the cell. Mainly, viral surface proteins have two functions: cell binding and membrane fusion. These functions can be combined in one protein or performed by different proteins.

Based on structural similarity, viral fusion proteins are divided into three main classes. The first class of fusion proteins includes surface proteins of virus families *Retroviridae* (human immunodeficiency virus, gp41) [6,7], *Filoviridae* (Ebola virus, GP2) [8], *Orthomyxoviridae* (influenza virus, HA) [9,10], *Paramyxoviridae* (parainfluenza, F-protein) [11], and *Coronaviridae* (coronaviruses, S-protein) [12]. These are homotrimeric formations consisting of three identical subunits. They contain α-helical structures and a fusion peptide located closer to the N-terminus and hidden in the middle of the protein trimer. The fusion mechanism for these proteins is similar and is implemented using heptad repeats (HR).

Fusion proteins of the second class are characteristic of the *Flaviviridae* family (E proteins of Denge virus, Zika virus, and yellow fever virus) and the *Bunyavirales* family (Hantaan and Puumala viruses) [3,4]. Fusion protein monomers consist of three main globular domains, which are predominantly composed of β-sheets, with the fusion peptide hidden in internal loops.

Unlike classes I and II of fusion proteins, the structure of type III fusion proteins consists of α-helices and β-sheets and includes an additional globular domain. The protein comprises of three protomers with a number of α-helices located in the center of the protein. Proteins of this class are characteristic of the families *Rhabdoviridae* (vesicular stomatitis virus, G protein), *Herpesviridae* (herpes simplex virus type I, gB protein), and *Baculoviridae* (baculovirus, gp64 protein) [2,3].

To fuse the viral and cellular membranes, significant internal conformational changes have to occur in most viral fusion proteins. Membrane fusion involves bringing two separate bilayers of cellular and viral membranes into close contact and then combining them [5]. Clearly, such a process proceeds by overcoming a high kinetic barrier, but from the point of view of thermodynamics, this process is valuable [4].

**Figure 1:**
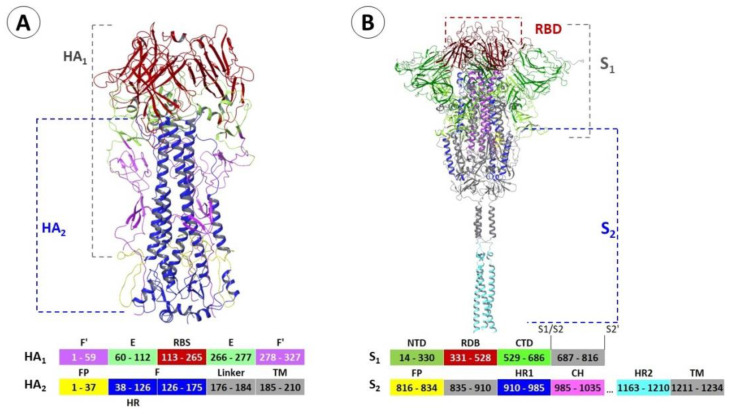
Structural features of the surface proteins of influenza virus and coronavirus according to X-ray diffraction analysis from Protein Data Bank [13]. Hemagglutinin (**A**) (amino acids numbering corresponds to PDB codes: 6Y5L and 1RU7 [10,14]): F’ (a. a. 1–59) and F’ (a. a. 278–327) are N- and C-terminal subdomains of HA_1_; E-domain [14] contains RBS—receptor-binding site, FP—fusion peptide, HR—heptad repeat, and F (a. a. 38–175) is a subdomain of HA_2_; TM—trans-membrane domain. S-protein (**B**): NTD—N-terminal domain; RBD—receptor-binding domain; CTD—C-terminal domain; S_1_/S_2_, S_2_′—sites of proteolysis; FP—fusion peptide; HR—heptad repeat, CH—central heptad; TM—trans-membrane domain.

Two surface proteins of influenza virus (A) and the spike protein of the SARS-CoV-2 (B) coronavirus are shown in Figure 1. Of course, the influenza virus and the coronaviruses belong to fundamentally different families of viruses. However, their surface proteins are fusion proteins of the first type. Both proteins consist of two subunits. In the first subunit, the receptor-binding site (in the case of HA) or domain (in the case of S-protein), whose key role is to bind to host cellular receptors, is localized. Heptad repeats of the second subunit are involved in structural rearrangements of proteins during the transition from pre- to post-fusion conformations, followed by the fusion of viral and cell membranes. Despite the noticeable differences between the structures of proteins, the presence of similar heptad repeats suggests that the mechanisms of fusion of viral and cell membranes for these viruses are similar.

**Figure 2:**
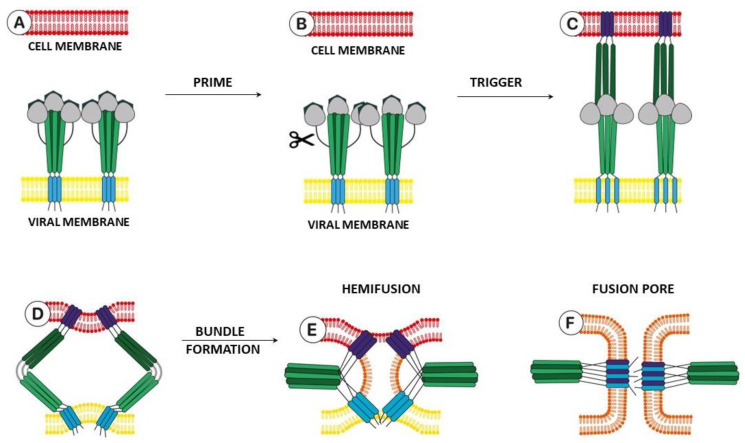
Model of the fusion process of viral and cellular membranes (adapted from [4]).

The model of the fusion process of viral and cellular membranes provided by class I fusion proteins is presented in Figure 2. Similar processes are also observed in class II and class III fusion proteins. The fusion protein is located on the surface of the viral envelope. Proteolytic cleavage or priming of a viral protein by a cellular protease is the first step (Figure 2, A→B) of the fusion mechanism that results in the opening of highly hydrophobic fusion peptides or fusion loops. In the case of class I proteins, the surface protein itself is subjected to proteolytic processing. For class II proteins, the heterodimeric partner protein “chaperone” is subjected to proteolytic cleavage [3,4]. A number of class III proteins can combine the features of the first two. However, for rhabdoviruses, whose fusion proteins belong to the class III, there is no obvious priming and most of the conformational transitions are reversible [3]. Priming transforms the protein into a metastable, i.e., thermodynamically unfavorable state B in expectation of an initiating process, for example, a decrease in the pH of the medium.

Moving from pre- to post-fusion is the next key step in the process (Figure 2, B→C). In the case of influenza virus haemagglutinin activation, the protonation of the inner space of virion is a trigger: conformational changes in the protein occur at a lower pH of the medium [10]. In the case of HIV, conformational rearrangements are initiated by binding to the CD4 receptor and CCR5 or CXCR4 co-receptors [7].

Conformational rearrangements in the protein lead to the formation of an intermediate structure called pre-hairpin structure C. Evidence for the formation of such a structure based on the example of influenza virus haemagglutinin is very strong [10]. In addition, studies of other types of fusion proteins suggest that this moderately long-lived intermediate C state is characteristic of most surface proteins, taking into account their structural features [3], including the SARS-CoV-2 S-protein [15].

Further, conformational rearrangements in the pre-hairpin C bring together the N- and C-ends of the heptads, attracting the viral and cell membranes to each other, contributing to finding the definition of metastable state D. The heptad formation forms a bundle of six helices, with the formation of the so-called “hairpin”, and, as a result, the cell and viral membranes reach a state of hemi-fusion E. Then the process continues until the complete membrane is formed with the formation of a fusion pore F, through which the genetic material of the virus penetrates into the host cells. At the same time, the structure of the hairpin trimer D is characteristic of all infections with viral fusion proteins [3,4,5]. This review presents the results of theoretical studies by molecular modeling methods aimed at finding new inhibitors of surface viral proteins, namely influenza virus haemagglutinin and the S-protein of human coronaviruses, including the SARS-CoV-2 strain. The following are the chapters on haemagglutinin of the influenza virus and coronavirus glycoprotein. In both cases, binding sites of known entry inhibitors are described, indicating the pharmacophore profiles of the site and functional amino acid residues. In the Supplementary materials of the review (Supplementary Table S1), a list of PDB codes corresponding to the crystallographic data of the geometric parameters of protein complexes with known ligands is presented. In addition, the structures of the compounds described in this review, in combination with their antiviral activity data and the site of binding to surface proteins, are summarized in Supplementary Table S2. The chapters on the features of model surface proteins (methods, approaches, and limits of their applicability) are presented in the supplementary materials.

## 2. Hemagglutinin of Influenza Virus

### 2.1. Structure and Function of Hemagglutinin

**Figure 3:**
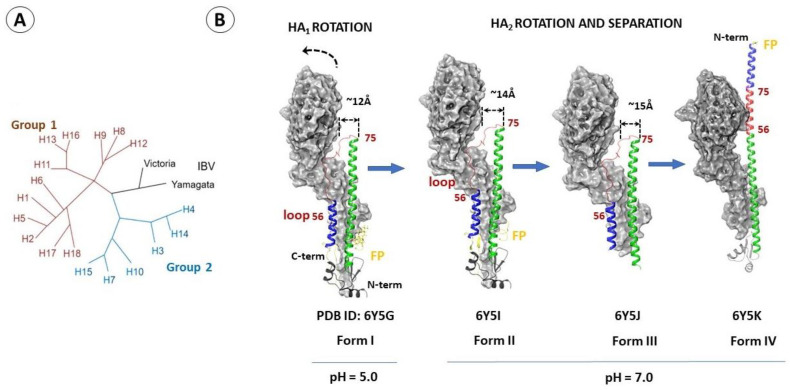
Hemagglutinin of the influenza virus. The phylogenetic tree (**A**). Conformational rearrangements of HA (**B**) the HA_1_ subunit is shown in gray; the short α-helix (a. a. 38–55) is highlighted in blue; the loop (a. a. 56–75) is red; the long α-helix (a. a. 76–126) is green; FP—fusion peptide is shown in yellow; the distances between the amino acids D_1_104 and R_2_76 are shown in Å.

Influenza virus hemagglutinin (HA) is a glycoprotein consisting of three identical subunits, each consisting of a variable HA_1_ globular domain binding to the cell receptor and a more conservative stem part of HA_2_ (Figure 1A). The key problem faced by the developers of new HA inhibitors is their high pleiomorphism [16]. Based on phylogenetic analysis, 18 antigenic subtypes of HA are described, which can be collected in 2 main groups [16,17,18]. Group 1 includes subtypes H1, H2, H5, H6, H8, H9, H11, H12, H13, H16, H17, and H18, while group 2 includes H3, H4, H7, H10, H14, and H15 (Figure 3A). In addition, there are two distinct classes of HA of influenza B viruses: the Yamagata-like and Victoria-like lineages [16]. These groups are structurally different in the regions involved in conformational rearrangements in the course of viral and cell membrane fusion [17,19].

The main function of HA is to ensure the penetration of the viral genome into the cytoplasm of the host cell. Penetration of the virus begins with the binding of the HA_1_ globular domain to the sialic acid (SA) receptor on the cell surface [20] followed by endocytosis. The acidic environment of the endosome starts the process of conformational rearrangements in the stem part of the HA_2_ domain, which leads further to the fusion of the viral and cell membranes (Figure 3B) [3,4,10,17,21,22]. As a result, the so-called fusion peptide is exposed to the outside, binds to the cell membrane, and fuses the viral and endosomal membranes. According to [10], this process proceeds through the formation of intermediate conformations (forms II–IV in Figure 3B), described individually by cryo-electron microscopy methods.

The process of membrane fusion is advantageous from the point of view of thermodynamics, but proceeds rather slowly due to kinetic difficulties [21,23]. It is assumed that when the pH decreases (Figure 3B of Form I), successive conformational rearrangements of HA are triggered [10,17].

At the first step, the subunit HA_1_ rotates, which leads to an increase of the distance between the centers of HA_1_ and HA_2_ (Figure 3B shows the distance between the amino acids D_1_104 and R_2_76) and weakening of the intermolecular contacts between the subunits (Figure 3B form II). In Form III, the distance between the domains continues to increase, accompanying conformational rearrangements in the HA_2_ domain. Next, the loop (the amino acid section 56–75 is highlighted in red in Figure 3B) turns by 180°, resulting in divergence of the helices. The fusion peptide at the N-end moves to become the N-end of the new α-helix (form IV) formed from an inverted short (shown in blue) and central α-helix (shown green in Figure 3B). Furthermore, the subunits HA_1_ and HA_2_, connected by a disulfide bridge, diverge and the membrane anchor is exposed [10,17,24]. According to the above observations, stabilization of the spiral loop–helix structure can be considered decisive in preventing the transition from form II to form IV [10].

### 2.2. Binding Sites of Small Molecules in HA_1_

**Figure 4:**
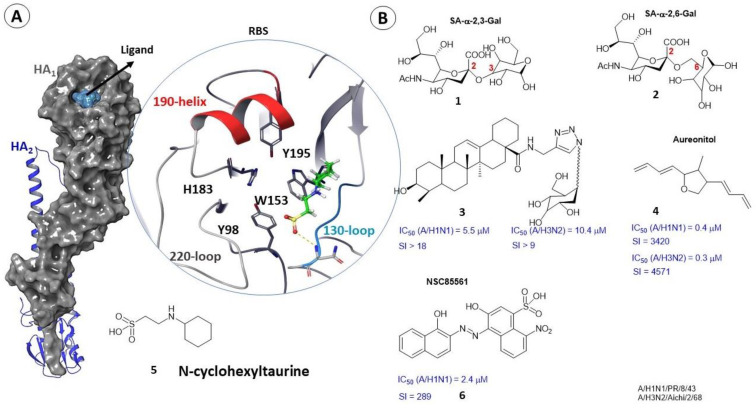
Protomer of HA. The location of *N*-cyclohexyltaurin **5** (**A**) (adapted from [25]) in the receptor-binding site. Inhibitors of RBS (**B**). SI—selectivity index.

The receptor-binding site (RBS) is localized in the variable globular domain of HA_1_. It is highly variable among 16 subtypes of influenza A virus [25]. The RBS is a shallow pocket located on the surface of the globular head of HA and it consists of amino acid residues 116–261. Four amino acids (Y_1_98, W_1_153, H_1_183 and Y_1_195) are conserved for all subtypes of HA except H17 and H18. Key amino acids, namely Y98 and W153, are located at the bottom of the binding pocket [26] and are surrounded by four structural elements: the 130-loop, the 150-loop, the 190-helix, and the 220-loop (Figure 4A). These elements are present in all HA subtypes, but their length and amino acid composition differ depending on the virus strain and are often key factors in the receptor recognition [25]. The mechanism of binding the key amino acids to sialic acids can be considered from the standpoint of molecular modeling [27,28]. A theoretical study by the methods of molecular dynamics showed that the amino acid Y_1_91 on the bottom of the binding pocket HA_1_ forms hydrogen bonds with α-2,3 or α-2,6 bound terminal SAs in various HA subtypes. However, the specificity of recognition may depend on the HA subtype [27]. Based on the fact that SA is an HA receptor, SA-based inhibitors can be used as potential agents against HA [18] (Figure 4). Unfortunately, creating antiviral drugs from sialic acid analogs has not been successful [16,29]. The reason is the very weak binding of the sialic acid receptor itself; the value of the dissociation constant is about 3–5 μM [29]. In addition, derivatives of monovalent SA (**1,2**) can hardly compete with native glycans [28,30]. Alternatively, polyvalent analogs of SA [31] or inhibitors that do not contain a carbohydrate residue can be considered. Such structures include, oleic acid conjugates (**3**) [32], aureonitol (**4**) [33], and small peptides [18] that can bind within the pocket of the RBS domain, for example. Molecular docking methods for most low molecular weight inhibitors assessed their affinity for RBS and described the nature of the intermolecular interactions.

To develop a potential RBS inhibitor, it would undoubtedly be efficient to use the crystal structure of HA in a complex with a native ligand. The non-commercial database Protein Data Bank [13] presents two HA complexes with a small molecule of *N*-cyclohexyltaurin (**5**) located in the receptor-binding pocket (Figure 4A). According to [29], the low molecular weight compound *N*-cyclohexyltaurin (**5**) mimics the binding of the natural sialic acid receptor with the receptor-binding domain NA_1_ due to the formation of similar hydrogen bonds and intermolecular interactions with the polar remnants of the 130- and 220-loops (Figure 4A). It is suggested in [29] that compound **1** can be used as a scaffold structure, and structural modifications of *N*-cyclohexyltaurin are recommended to fill the binding pocket more tightly, thus increasing its affinity. This compound is also interesting because it can also be bound in the stem part of the HA_2_ domain. In other words, it can prevent HA binding to sialic acid receptors and force additional structural restrictions on the fusogenic transitions of the protein. In [34], molecular modeling techniques in conjunction with biological experiments were used to search for potential HA_1_ inhibitors. Based on the results of a theoretical assessment of the affinity of more than 200 compounds to the sialic acid binding site, the authors chose the lead compound NSC85561 (**6**) (Figure 4B). Further biological experiments to evaluate IC_50_ confirmed the results of molecular modeling.

Despite the fact that the first role of HA entails its binding to a cell receptor, the region close to RBS is also attractive for studying the interaction of antibodies with HA_1_, in particular, of influenza viruses of different strains. Thus, in [35] the methods in silico estimated the affinity of a number of antibodies to potential binding sites in the globular domain of HA. Similar studies [36] are of greater interest from the perspective of the rational design of a universal vaccine. However, for drug development, in the case of HA, binding sites located in the conservative region of the stem part of the protein are most often considered [37,38,39,40].

### 2.3. Binding Sites of Small Molecules in HA_2_

The development of numerous small molecule inhibitors of HA, aimed at blocking the fusion mechanism of viral and cell membranes, began at the end of the 20th century [16]. As a rule, experimental methods allowed an assessment of the antiviral activity of compounds and the substantiation of a potential biological target. However, the place of binding of potential HA inhibitors until this time was a mystery, and now it is often a controversial issue.

In fact, finding potential binding sites in the stem part of HA by molecular modeling methods is quite a difficult task, certainly requiring experimental evaluation. The lack of crystalline HA complexes with potential inhibitors significantly complicated the development of new drugs. It can be assumed that one of the first recorded crystals [17] with a small molecule of tert-butyl hydroquinone (TBHQ) opened up the possibility for researchers to conduct theoretical calculations in order to search for compounds whose binding in the cavities of the second subunit of HA_2_ can lead to inhibition of the fusion of viral and cell membranes.

#### 2.3.1. Binding Site of TBQH and Umifenovir

**Figure 5:**
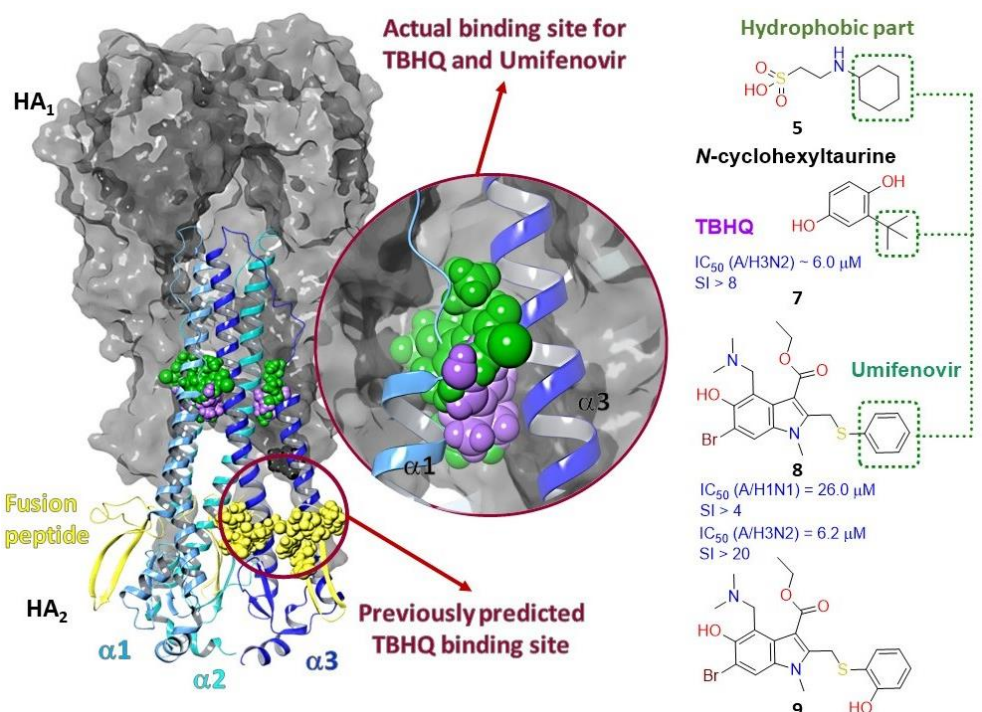
Binding sites of HA inhibitors.

The first attempts to describe the binding site of potential HA inhibitors were made at the end of the 20th century. In 1993 and in [41], based on the results of molecular docking, it was suggested that the site of binding tert-butyl hydroquinone (TBQH) (**7**) is located at the site of the fusion peptide HA_2_ (the secondary structure is colored yellow in Figure 5). The hydrophobic cavity of the binding site is surrounded by amino acids 4, 7–19, 24, 25 of HA_2_, and 17, 325 of HA_1_ (amino acids in Figure 5 are represented as yellow spheres). In 1997, Hoffman et al. [42] estimated the affinity of small molecules to the potential binding site described in [41] including the model compound TBHQ. However, the description of the crystal structure of HA with TBHQ [17] refuted the assumption [41]. According to electron density maps, TBQH binds to HA at the interface between two trimer protomers. In other words, three molecules of TBHQ can bind to one HA trimer. Binding sites of TBHQ are formed by the residue of the long α-helix of one protomer and the short α-helix of a neighboring protomer (Figure 5).

Interactions of TBHQ with HA are mostly of hydrophobic nature, as the binding site is saturated with hydrophobic amino acids: L_1_29, L_2_98, and A_2_101 of one protomer and L_2_55 and L_2_99 on another one. In addition, compound **7** forms contact with ionized amino acids R_2_54, E_2_57, and E_2_97. Then, the hypothetical inhibitory mechanism of TBHQ action is to increase the stability of the complex. According to [17], the described hydrophobic binding site is formed in only one of two phylogenetic groups of HA, and crystal complexes were recorded only for strains H14N5 and H3N2, i.e., for HA of the second group.

TBHQ and Umifenovir, sold under the name Arbidol (**8**) which is an antiviral drug approved in Russia and China in clinical practice [43], stabilize the pre-fusion conformation of HA. The molecule binds between two α-helices of different protomers [44] and inhibits important conformational rearrangements associated with membrane fusion at low endosomal pH (Figure 5). Wright and co-authors [45] carried out a number of structural modifications of Umifenovir and obtained its structural analog (**9**), whose affinity to the potential binding site of TBHQ and Arbidol is an order of magnitude higher than the value characteristic of the latter. As mentioned above, according to [29], *N*-cyclohexyltaurin can bind to HA_1_ subunits at the receptor-binding site and in the stem portion of the HA_2_ domain between the short and long α-helixes of different protomers. Kadam and Wilson drew attention to the hydrophobic fragment of this compound, cyclohexyl, identifying it as an analog of the hydrophobic fragments (Figure 5) of tert-butyl in TBHQ and the aromatic ring in Umifenovir, which are exposed to the same hydrophobic amino acids: L_1_29, L_2_98, A_2_101, L_2_55, and L_2_99.

**Figure 6:**
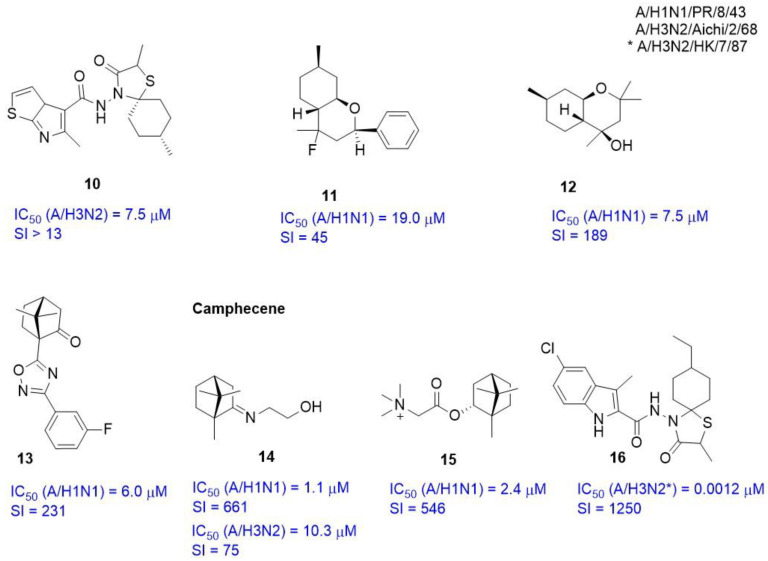
Inhibitors of HA binding in the TBHQ binding site.

The crystallographic structures of complexes HA-TBHQ (PDB code 3EYM) and HA-Umifenovir (PDB codes 5T6N and 5T6S) formed the basis for theoretical calculations by molecular docking methods to assess the affinity of inhibitors (**10–16**) presented in Figure 6 to the TBHQ/Umifenovir = binding site.

Based on the results of molecular modeling, together with biological experimental data, the mechanism of antiviral action with a number of compounds was described: spiro-heterocyclic compounds **10** [46], isopulegol-derived substituted octahydro-2Hchromen-4-ols (**11**, **12**) [47,48], O–acylated amidoximes and substituted 1,2,4–oxadiazoles (**13**) [49], camphecene (**14**) [50] and its analogs [51], a quaternary salt based on (-)-borneol (**15**) [52], and the spirothiazolidinone derivatives of indole (**16**) [53]. Authors used the molecular modeling methods in all these cases. They considered a hydrophobic cavity enclosed between two α-helixes of different protomers of HA (or TBHQ site) as a potential binding site. The high potential of natural terpene compounds as effective anti-viral agents should be noted [54].

#### 2.3.2. Epitopes of HA as a Possible Binding Site for Inhibitors

**Figure 7:**
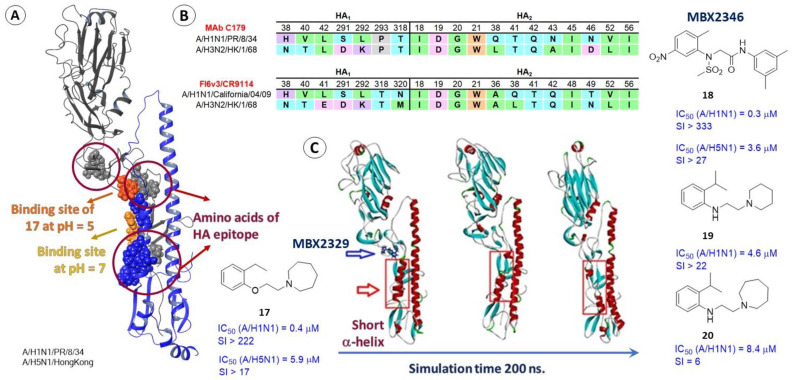
Epitopes of HA as a possible binding site for HA inhibitors. The binding site of MBX2329 (**A**): the yellow and orange spheres show the location of the compound at different pH values of the medium and the amino acids of the HA epitope are shown in gray (correspond to HA_1_) and blue (correspond to HA_2_) spheres. The table (**B**) shows the contact between amino acids of HA and residues of antibodies MAb C179, CR9114, and FI6v3 form significant intermolecular interactions. The figure from the original paper [37] (**C**), where a noticeable effect of MBX2329 on the secondary structure of HA (especially on the short protomer α-helix) is visualized during 200 ns of molecular dynamic simulations.

The stage of influenza virus entry into a cell can be blocked by broad-spectrum neutralizing antibodies that bind to HA epitopes [55,56] including those located on the stem part of the HA_2_ subunit. Various antibodies, e.g., MAb C179, CR9114, and FI6v3, come into contact with the protein surface at the interface of two subunits, forming intermolecular contacts with amino acids on the HA_1_ 38–41, 291–293, and 318–320 and on the HA_2_ side 18–21 and 36, 38, 41, 42, 48, 49, 52, and 56 (Figure 7A,B). These amino acids can be considered as functional and the site of contact of HA with antibodies is the site of potential binding of HA inhibitors. Thus, in [57] the contact region of HA and antibody MAb C179 was considered as a potential binding site for two promising compounds: MBX2329 (**17**) and MBX2546 (**18**).

These compounds (**17** and **18**) were selected from more than 106,000 chemical structures based on the results of high-throughput screening using a lentivirus-based pseudoviral system with HA on its surface. Compounds exhibit inhibitory activity against a number of strains of influenza virus in micromolar concentrations, IC_50_ values fall in the range from 0.30 to 3.60 μM depending on the strain of the virus. The paper [57] suggests that MBX2329 and MBX2546 bind to the stem region of HA_2_ and lead to disruption of the fusion process. According to NMR analysis, the binding of these compounds to HA forms a series of key contacts between atoms of these compounds and amino acids of the first and second subunits.

Subsequently, in [37] large-scale theoretical studies were carried out using molecular dynamics and showed that the most likely binding site of MBX2329 at pH = 7 is located at the border of two subunits in a hydrophobic pocket surrounded by side chains V_1_31, L_1_290, T_1_316, I_2_47, T_2_48, and V_2_51. It is noteworthy that when simulating the interaction of the ligand with the HA surface at a reduced pH, its estimated binding site is slightly higher (the molecule is shown in orange). At the same time, according to the results of molecular dynamics [37], the compound is likely to have a significant effect on the secondary structure of HA (Figure 7C), namely the short α-helix.

Large-scale theoretical calculations conducted using methods of molecular dynamics allowed the authors of [37] to describe the possible mechanism of the inhibitory action of MBX2329. The module of heptad repeats of HA plays a key role in conformational rearrangements, in which the loop connecting the short and long α-helices changes its secondary structure and leads to the formation of one α-helix. To control this transition, water molecules have to interact directly with hydrophilic amino acid residues [37,58]. Then, the main inhibitory effect of agent **17** is attributed to it stabilizing the bonds of two subunits and preventing water molecules from entering the HA. Interestingly, the paper [59] describes inhibitors of HA **19** and **20** (Figure 7) as compounds similar in their structural and pharmacophoric descriptors to substance MBX2329. Compounds **19** and **20** are active against influenza virus strain A/H1N1/PR/8/34 in micromolar concentrations. It is logical to assume that the binding of these compounds to HA should occur at the epitope site, as occurs with agent **17**. However, the paper [59] suggests that inhibitors **19** and **20** bind in the TBHQ site (Figure 5). The influenza virus strain resistant to **19** contains the amino acid substitutions T_2_107I and R_2_153I. Authors of [59] believe that the T_2_107I mutation is the most significant and is located in a cavity close to the TBHQ site. The results of molecular modeling (docking and molecular dynamics) show that the studied compound can bind in the TBHQ site to form intermolecular interactions with the same residues. Why the authors did not consider alternative binding options remains a mystery.

Based on the crystal structures of complexes HA with FI6v3 and CR9114, small cyclic peptides were developed [60]. New peptides exhibit nanomolar activity by binding to a highly conserved stem epitope and blocking conformational rearrangements of HA. Crystal structures of peptide complexes with HA of the A/Puerto Rico/8/1934 virus strain (H1N1) are presented in the Protein Data Bank (a list of PDB codes is presented in SM-Table S1).

**Figure 8:**
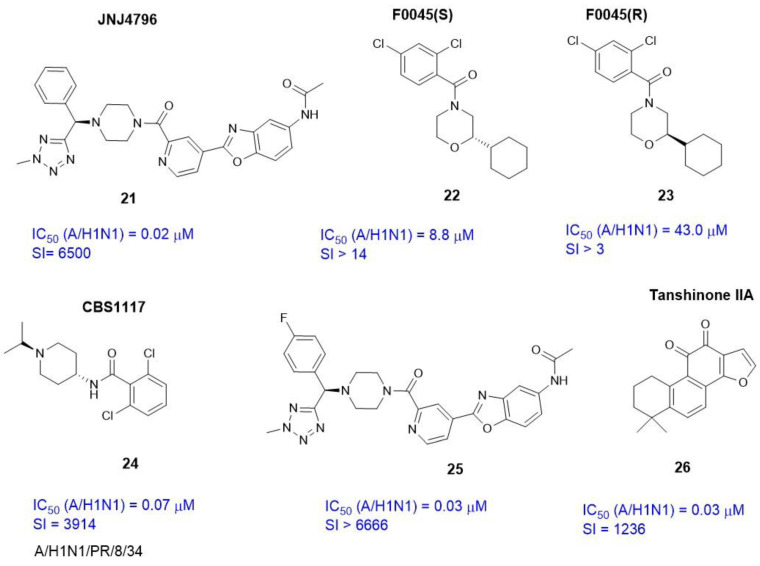
Inhibitors of HA binding in the HA epitopes.

In 2009, Ekiert and co-workers [61] described the antibody bnAb CR6261. The antibody binds to the surface of the stem part of HA and neutralizes most of the influenza A viruses. The presence of the crystal complex HA-CR 6261 (PBD code 3GBN) inspired the authors of [62] to develop a low molecular weight inhibitor JNJ4796 (**21**) (Figure 8). The main idea of creating compound **21** was to search for small molecules that mimic the binding of CR6261 to the surface of HA. Authors of [62] screened about 500,000 low-molecular compounds that selectively targeted the CR6261 epitope on HA. As a result, within the benzylpiperazine class, the active compound JNJ4796 (**21**) was identified.

An epitope recognized by the small molecule **21** was similar to the epitopes associated with bnAb, namely CR6261, FI6v3, and CR9114. In other words, agent **21** binds in a hydrophobic pocket on the outer surface of HA (H1) and mimics CR6261-like intermolecular interactions with amino acids: H_1_18, H_1_38, L_1_42, T_1_318, G_2_20, W_2_21, T_2_41, and L_2_56. Thus, the mechanism of antiviral action of **21** is to inhibit pH-sensitive conformational rearrangements that are triggered by the fusion of viral and cell membranes. The compound exhibits antiviral activity against influenza A virus strains at nanomolar concentrations (Figure 8).

The crystal structures of the complexes of HA with ligands **21**–**24** formed the basis for the development of new antivirals, namely potential inhibitors of HA. Thus, the paper of [63] describes temporins which are small peptides that presumably bind within the region of HA in contact with the antibodies. Molecular modeling to assess the affinity of temporins to the binding site was carried out on the basis of crystalline structures of small peptides with HA described in [60].

The design and application of a fluorescent polarization (FP) probe based on P7 peptide allowed the authors of [64] to conduct high-throughput screening (HTS) of 72,000 compounds and identify a new low molecular weight molecule F0045(S) (**22**) with high affinity for the stem epitope HA H1N1 (Figure 8). The crystal structure of the HA-**22** complex (PDB code 6WCR) was recorded. Interestingly, the R-stereoisomer F0045(R) (**23**) is characterized by less pronounced antiviral activity. The authors of [64] associate such selective activity of stereoisomers with different locations of the aromatic ring in the hydrophobic pocket of the HA epitope.

The binding region of CBS1117 (**24**) [65] with H5 HA was described using methods of X-ray crystallography, NMR, and experiments using site-directed mutagenesis. Compound **24** binds on the protein surface and forms a number of intermolecular interactions with amino acids that play a key role in binding to antibodies.

The authors of [66] performed a structural modification of agent JNJ4796 (**21**) and synthesized a number of analogs containing substituents in the aromatic ring. Based on a number of biological tests, the leader-4-fluorine derivative compound was selected (**25**) (Figure 8). Compound **25** exhibits antiviral activity against influenza strain A/H1N1 that is commensurate with the activity of agent **21**. At the same time, the introduction of a fluorine atom into position 4 of the aromatic ring **21** leads to a decrease in the cytotoxicity of the new compound **25**, and as a result, to an increase in the selectivity index. For molecular docking, authors of [66] used the crystal structure of the HA-JNJ 4796 (**21**) complex with an estimated affinity of **25** to the binding site and described an additional hydrophobic interaction between the fluorine atom (**25**) and V_2_18.

According to [67], Tanshinone IIA(**26**) (Figure 8), the biologically active compound isolated from redroot sage (*Salvia miltiorrhiza*) exhibits pronounced activity against influenza virus A/H1N1. The affinity of **26** to the binding site of HA was evaluated by molecular docking and molecular dynamics. F0045 (S) and the crystal structure of HA complex with F0045 (PDB code 6WCR) were considered as a reference compound in theoretical calculations.

#### 2.3.3. Alternate Binding Sites

**Figure 9:**
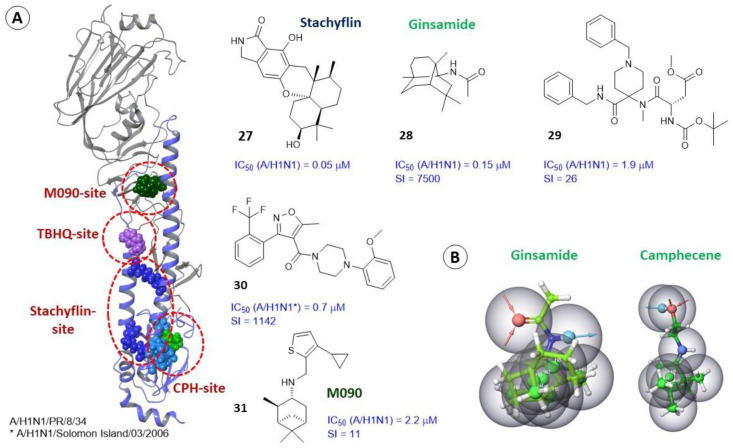
Binding sites of HA inhibitors. The binding sites of stachyflin, camphecene, and M090 (**A**). The pharmacophoric profiles of camphecene and ginsamide (**B**) hydrophobic regions of the molecule are shown in green and donor and acceptor regions are in blue and red, respectively. The protonated nitrogen atom is shown in blue.

In some cases, compounds exhibiting pronounced antiviral activity against influenza and exhibiting inhibitory activity against HA can bind in alternative binding sites other than the location of previously discussed TBHQ and Umifenovir, as well as from the sites of contact of antibodies with protein surfaces. Thus, in [68] the authors described the antiviral activity of natural metabolite stachyflin (**27**) against a number of strains of the influenza virus (Figure 9). Stachyflin inhibits the replication of viruses of different strains, such as A/Puerto Rico/8/1934 (H1N1), A/Narita/1/2009 (H1N1) pdm, A/Singapore/1/1957 (H2N2), A/duck/Hokkaido/5/1977 (H3N2), A/Hong Kong/483/1997 (H5N1), A/turkey/Italy/4580/1999 (H7N1), and others. The antiviral activity of **27** was tested against various strains of the virus corresponding to 14 types of HA. After selecting and sequencing the stachyflin-resistant strain (A/WSN/1933 (H1N1), A/Puerto Rico/8/1934 (H1N1), A/chicken/Ibaraki/1/2005 (H5N2), and A/chicken/Taiwan/A703-1/2008 (H5N2)), a substitution of amino acid residues in the α-helices of the HA_2_ subunit, was detected. Molecular modeling allowed authors to describe the probable Stachyflin-binding site. A small cavity is located between two α-helices of one protomer HA_2_. In a resistant strain of the virus, the amino acids D_2_37, L_2_51, T_2_107, and L_2_121 are replaced, and they are the key in the binding site (Figure 9A), forming a series of intermolecular interactions with the compound under study. The proposed mechanism of inhibitory action of agent **27** is as follows: the molecule is located between two α-helices (short and long) forming a series of non-covalent interactions with amino acid residues and, as a result, keeping the α-helices in a compressed state. The binding of stachyflin increases the energy barrier required for this conformational transition and the formation of one α-helix.

New antiviral amino derivatives based on (+)-camphor are described in [50,69] in which camphecene (**14**) or CPH (1,7,7-trimethylbicyclo [2.2.1] heptane-2-ilidene-aminoethanol) was identified as a lead compound (Figure 6). Camphecene demonstrates high inhibitory activity against a number of strains of influenza virus, including rimantadine-resistant ones. Numerous biological experiments have shown a wide range of its anti-influenza activity at low concentrations, as well as very low toxicity. In addition, there is experimental evidence that camphecene reduces the fusogenic activity of HA. HA was considered as a biological target (Figure 9A) based on the presence of a hydrophobic fragment in camphecene similar in pharmacophoric profile to fragments of already known inhibitors of HA TBHQ and Umifenovir. Firstly, the location of TBQH/Umifenovir (or TBHQ site) was considered as a potential site for the binding of camphecene. According to the results of molecular docking, camphecene shows affinity to this site commensurate with the data characteristic of reference inhibitors. In order to confirm the mechanism of antiviral action, the work in [70] described a camphecene-resistant influenza virus obtained as a result of the propagation of influenza A/H1N1 virus for 6 passages in the presence of increasing concentrations of the drug. Sequencing of the the HA gene of the camphecene-resistant influenza virus showed the presence of V_2_115L amino acid substitution (the numbering of amino acids corresponds to the PBD code 4LXV [71], the original article uses the numbering of the PBD code 1RU7 [14]) in the stem portion of hemagglutinin in the HA_2_ subunit. Molecular modeling showed that there is a small hydrophobic cavity at the site of proteolysis of HA_2_ (Figure 9A) where the camphecene molecule can be embedded, thus forming hydrogen bridges with V_2_115 and I_1_9. Replacing valine with leucine leads to a reduction in the size of the cavity, which affects the decrease in affinity of camphene to this binding site. It is extremely important to note that the resulting mutants are characterized by a significant decrease in virulence and pathogenicity for animals. Such a remarkable result seems to be associated with the peculiarities of hemagglutinin functioning, specifically with its interaction with cellular proteases.

Another compound, ginsamide (**28**) [72], exhibiting pronounced activity against influenza virus can bind at the CPH site by contacting V_2_115. This binding site was selected based on the similarity of the pharmacophoric profiles of camphecene and ginsamide (Figure 9B) as well as based on the result of sequencing a ginsamide-resistant influenza A virus. Serial passages of the influenza virus in the presence of ginsamide resulted in the selection of the V_2_115L mutation in HA_2_. In other words, both camphecene [70] and ginsamide [72] can induce virus resistance using the same mutation in HA_2_.

Interestingly, [73] describes a hydrophobic cavity surrounded by amino acid residues K_2_123, E_2_120, Y_2_119, and F_1_9 which are located close to V_2_115 (Figure 9A). Based on the data obtained by molecular docking and molecular dynamics methods, it is assumed that the compound (**29**) (Figure 9) will exhibit its antiviral activity precisely by binding at the described binding site or at the camphecene-binding site. The presence of a hydrophobic cavity at the site of proteolysis that is suitable for binding small molecules was mentioned earlier in [70]. However, [73] describes this site as a fundamentally new binding site for potential HA inhibitors.

In [74], new compounds active against the influenza virus were identified. Based on a number of biological experiments, Kim and co-authors identified one leading compound IY7640 (**30**). The molecular target of agent **30** is the highly conserved stem region of HA. Using molecular docking methods, the authors searched for a potential binding site of substance **30** considering the HA epitopes and the TBHQ site. Agent **30** binds between two key α-helixes close to the viral membrane.

Finally, it is necessary to mention another binding site of HA inhibitors on the example of substance M090 (**31**), located between the long α-helix and the loop connecting the short and long α-helixes [75]. The binding of M090 (Figure 9) can prevent a transition in which two α-spirals turn into one. The binding of **31** was also predicted on the basis of molecular modeling.

The selection of the binding site and inhibitors of HA is one of the most difficult tasks of molecular modeling in the development of anti-influenza drugs. The presence of crystal structures of HA complexes with ligands and probable inhibitors facilitates the task of researchers greatly. However, here it is necessary to consider the difference in structural descriptors and pharmacophoric profiles of the studied compounds.

Typically, in most scientific publications describing alternative binding sites, molecular modeling methods are presented in conjunction with data from physical and biological experiments, such as NMR studies and/or mutagenesis. Of course, to confirm the alternative locations of potential HA inhibitors, it is desirable to have a crystal structure of the HA-inhibitor complex.

### 2.4. Differences in the Binding Sites of HA_2_ of Different Phylogenetic Groups

The antiviral activity of most known HA inhibitors is evaluated against different strains of the influenza virus. A number of the studied compounds show activity against strains of influenza belonging to either the first or the second HA types. Thus, spirocyclic derivatives **10** [46] show pronounced activity against the A/H3N2 virus and are not active against A/H1N1. In contrast, agent **27** [68] is active against strains that belong to the first group of HA: IC_50_(H1) = 0.05–1.95 μM, IC_50_(H2) = 0.16 μM, IC_50_(H5) = 0.17–4.70 μM, and IC_50_(H6) = 0.44–0.65 μM; however, IC_50_ values against viruses bearing HA of the second group (H3, H4, and H7–H16) were above 6.50 μM. This compound [74] inhibits influenza viruses of A/H1N1, A/H1N1pdm09, A/H5N1, and A/H6N2 subtypes at concentrations of 0.7 up to 59.6 μM, while A/H3N2 and A/H7N9 viruses were inhibited with IC_50′_s of 83.0–221.0 μM. Clearly, this selective activity of HA inhibitors is most likely related to the structural features of the protein. Despite conservatism of the stem part of the HA domain, the amino acids surrounding the described binding sites may differ.

For example, in [17] when describing the binding site of TBHQ, it is assumed that such a hydrophobic region can only be found in HA_2_ of the second phylogenetic group. The crystal structures of HA with TBHQ and Umifenovir are recorded for H3, H7, and H14 subtypes. In fact, in [45] it is shown that Umifenovir binds with higher affinity to HA of the second group (K_D_ = 5.6–7.9 μM) than with HA of the first group (K_D_ = 18.8–44.3 μM). In [40], molecular modeling methods evaluated the affinity of Umifenovir to various binding sites in HA. The paper notes that Umifenovir can interact with all subtypes of HA, but with different affinities. Umifenovir shows the greatest affinity to the H7 binding site.

In a recently published paper [49], the authors compared the amino acid sequences of the binding sites for M090, TBHQ, and CPH for influenza viruses of H1 and H7 subtypes. Although the pharmacophoric profiles of the described binding sites are similar, in all cases hydrophobic amino acids predominate. Indeed, a number of a. a. substitutions are observed. In this case, it can be expected that the affinity of potential inhibitors to the binding sites of different types may differ [49,52]. Investigations [68,74] also take into account the difference in amino acid residues of potential binding sites of substances **27** and **30**, which explains their different antiviral activity against different subtypes of influenza virus.

Based on the analysis of the literature data, it can be noted that HA inhibitors are characterized by different structural descriptors and different pharmacophoric profiles. It is very difficult to divide them into any groups and associate their structure with the place of binding in the HA. Moreover, inhibitors vary even in size and molecular weight. One would assume that small molecules, such as substances **11**–**15** (Figure 6), should bind only in small hydrophobic pockets, such as the TBHQ or CPH site, while large molecules such as JNJ4796 can bind to the surface of the HA on the epitope side. However, it is not that simple. Small molecules such as **17**, **18**, **22**, **23**, **24**, and **26** (Figure 8) inhibit the function of HA by binding to the epitope surface, and bigger molecules, namely **8**–**10**, **16**, and **29** (Figure 5, Figure 6 and Figure 8), can localize to the stem part of the protein, thus preventing conformational rearrangements. Objectively, the search for new inhibitors of entry is impossible without understanding the mechanism of HA action. It is necessary to clearly understand what specific conformational rearrangements occur during the transition from pre- to post-fusion conformation and when it occurs. Fortunately, this process is well studied and described. In addition, many scientific publications and, of course, the presence of geometric parameters of HA complexes with various ligands greatly help in solving the problem. Moreover, the use of molecular modeling methods allows one not only to predict the binding site or describe the mechanism of antiviral action, but also to substantiate the biological properties of the virus carrying certain amino acid substitutions. Such properties include, for example, the pathogenicity of the virus, the spectrum of target cells, sensitivity or resistance to potential or used inhibitors, etc.

## 3. Glycoprotein (Spike Protein) of Coronaviruses

### 3.1. Structure and Function of the Spike Protein

Surface S-glycoprotein is a type I transmembrane fusion protein 180 to 200 kDa in molecular weight. The N-terminus of the protein faces the extracellular space; it is held in the viral membrane through a transmembrane domain with a short C-terminal segment facing the intracellular space (Figure 1). The S-protein of coronavirus plays an important role in the life cycle of the virus: it regulates virus binding to the surface receptor, penetration into the host cell, and it is the main target for the immune response of the host. In the viral membrane, the protein is involved in two important events: binding to the cell receptor and the subsequent fusion of viral and cell membranes. Two possible options for the entry of the virus into the cell are described: the so-called early and late entry [76]. After binding to the cell receptor, the S-protein is activated using free or membrane-bound proteases (e.g., transmembrane serine protease type 2, TMPRSS2). In the endosome, at reduced pH, cathepsin L (protease) is activated, which cleaves the S2’ site and starts the fusion process of viral and cell membranes, thereby releasing the virus genome into the cytosol.

**Figure 10:**
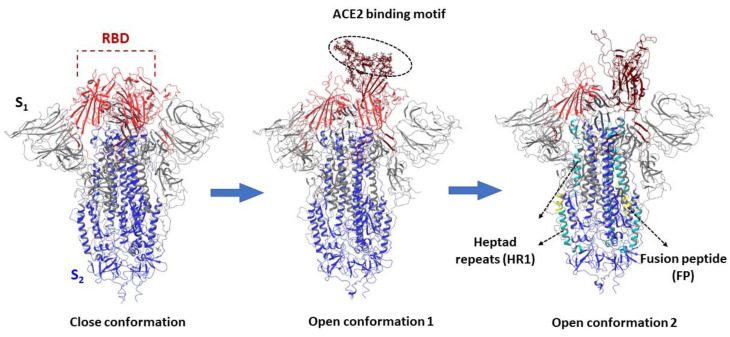
Sequential opening of RBD in the “bulb” head of the S-protein of SARS-CoV-2. The secondary structures of subunits S_1_ and S_2_ are shown in gray and blue, respectively, and the RBD in red. In the open conformation, two heptad repeats (HR1, a. a. 910–985) are highlighted in light blue. The fusion peptide (FP, a. a. 816–834) is shown as a yellow secondary structure.

Visualization of the S-protein monomers of the coronavirus shows that the subunits S_1_ and S_2_ form the “bulb” consisting of the head and stem region, respectively. The S_1_ subunit contains two subdomains, an N-terminal domain (NTD) and a C-terminal domain (CTD). In different coronaviruses, fragments of one or both subdomains can form a receptor-binding domain (RBD). According to currently available information on the high-resolution crystal structure [76,77,78,79], the RBD moves in the same manner as a hinge between two conformations (“up” or “down”), exposing the amino acid sequences of the binding motif to angiotensin-converting enzyme 2 (ACE2). At the same time, according to crystallographic data [79], the disclosure of the RBD occurs sequentially (Figure 10).

The transmembrane part of S_2_ contains domains involved in the fusion of the viral and cell membranes. These are the fusion peptide and two heptad repeats HR1 and HR2 (Figure 1 and Figure 10). The HR domains consist of α-helices and, as a rule, their position and amino acid sequence for the entire family of coronaviruses is conservative. S-protein of SARS-CoV-2, similarly to haemagglutinin of influenza virus, refers to fusion proteins of I type, which is due to the structural characteristics of its fusion domain and the need for splitting by the protease. The fusion reaction of the viral and cell membranes, catalyzed by the S-protein of the coronavirus, proceeds through the same successive states as for other proteins of the first type.

### 3.2. Small Molecule Binding Sites in S_1_

Coronaviruses use a wide range of receptors to enter target cells. Despite the highly conservative amino acid sequences in S_1_, S-proteins of various coronaviruses penetrate the cell binding to various receptors. Hence, epidemiologically important human coronaviruses HCoV interact with N-aminopeptidase (CD13) or with *N*-acetyl-9-O-acetylneuraminic acid located on epithelial host cells. MERS-CoV penetrates into the cell by interacting with dipeptidyl peptidase DPP-IV; SARS-CoV and SARS-CoV-2 bind to angiotensin-converting enzyme 2 (ACE2) [80]. Along with binding to a cellular enzyme, SARS-CoV-2 can bind other surface proteins to enter the host cell [12]. Neuropilin-1 is expressed in neurons and provides virus penetration by binding to the cleaved form of the S-protein [81]. In addition, the surface protein SARS-CoV-2 is able to bind to the CD147 receptor, which also mediates virus penetration into the cell [82].

### 3.3. Binding Site in the Receptor-Binding Domain of S_1_

**Figure 11:**
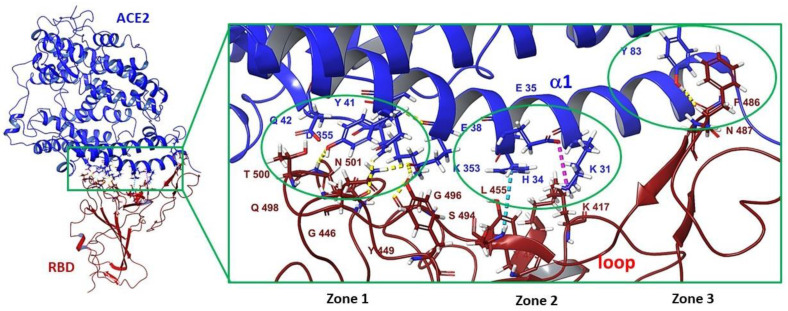
Interface of the receptor-binding S-protein of SARS-CoV-2 coronavirus with ACE2: hydrogen bonds, salt bridges, and pi–pi stacking interactions are shown in yellow, purple, and blue intermittent lines, respectively.

The receptor-binding domain (RBD) of the surface protein SARS-CoV-2 binds with high affinity to the cellular enzyme ACE2. This fact may indicate that interference with the RBD–ACE2 binding interface can potentially reduce the risk of infection [83] The crystal structure of the RBD–ACE2 complex is described in some detail in [84,85]. The RBD loop is in contact with the arcuate helix of the proteolytic domain of enzyme ACE2. The domain–enzyme-binding interface is divided into three contact zones where amino acid residues on both sides form various intermolecular interactions (Figure 9). The first contact zone (zone 1) is located on the side of the N-terminus, where the amino acids of RBD Q_1_498, T_1_500, and N_1_501 form hydrogen bridges (shown in Figure 11 in yellow lines) with amino acid residues of enzyme Y41, E42, K353, and R357. The central part of enzyme α-helix and the domain loops are contacted by forming a salt bridge (purple dotted line) between K_1_417 of the receptor-binding domain of the S-protein and K31 of ACE2 and π-π stacking contact (blue dotted line) between aromatic rings Y_1_453 (RBD) and H34 (ACE2). At the C-terminus (contact zone 3), N_1_487 of RBD contacts Y83 enzyme, and F_1_486 forms a van der Waals interaction with M82 [83].

**Figure 12:**
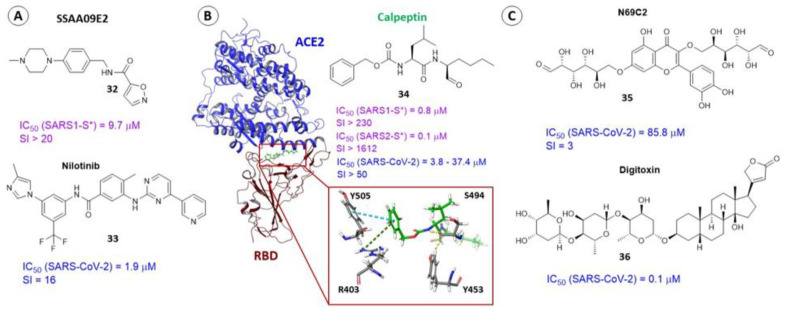
Inhibitors of S_1_ SARS-CoV-2. Molecular structures (**A**–**C**). The location of calpeptin (**B**) in the RBD–ACE2 interface: hydrogen bonds are shown by yellow dotted lines, π–π and π–cation stacking interactions are indicated by blue and green dotted lines, respectively. *-Pseudotype-based assay.

In 2013, the authors of [86] selected three leaders among 3000 compounds based on high-throughput screening using a pseudoviral system. The molecule of SSAA09E2 (**32**) (Figure 12A) inhibits the binding of the surface protein of SARS-CoV-1 to ACE-2 in micromolar concentrations. A logical continuation of the story about agent **32** is [87], in which affinity of substance **32** and nilotinib (**33**) to the “most pharmacologically dangerous pocket” located at the interface between RBD of SARS-CoV-2 and ACE2 was estimated using molecular modeling methods. The results of molecular docking show a high affinity of these compounds to the site of contact of the domain and the enzyme. According to the authors, binding of agents **32** and **33** (Figure 12A) to RBD could potentially interfere with some important intermolecular interactions between amino acids RBD and ACE2, namely Y_1_453 and H34, Q_1_493 and E35, and Y_1_449 and D38. Furthermore, if agent **33** does indeed show activity against SARS-CoV-2 at concentrations of 1.56–2.60 μM [88,89]), there are no published data confirming the activity of substance **32** against SARS-CoV-2. In addition, in [90] authors used fluorescent and magnetically modulated biosensors to develop a rapid and sensitive tool for screening inhibitors of S-protein and ACE2 interactions and showed that even at the highest concentration of compound **32** in 100 μM, inhibition of interaction of RBD of SARS-CoV-2 S-protein and ACE2 does not occur.

In [87,91], based on molecular modeling data, it is shown that the biological target for substance **33** is exactly the spike protein, while in [89] compound **33** is considered an inhibitor of the main protease M^PRO^. The results of MD calculations show a high affinity of agent **33** to the M^PRO^ catalytic site. Moreover, in [92] virtual screening of eight compounds, including **33** (Figure 12A), was carried out to estimate the affinity to the NSP12–NSP7–NSP8 complex necessary for replication and transcription of the virus. The authors also note that all the studied compounds bind well to the complex and are recommended as candidates for the treatment of coronavirus infection. Unfortunately, the entire history of searching for information about active inhibitors of SARS-CoV-2 entry is full of such contradictions. At the same time, we cannot deny the multi-targeting of agent **33** and the possibility of its synergistic effect against SARS-CoV-2. It should be noted that tyrosine kinase inhibitors, to which substance **33** belongs, have shown their activity in clinical practice [93,94].

The authors of [95] carried out high-throughput screening (HTS) of an extensive library of drugs that can be repurposed to find potential therapeutic agents against SARS-CoV-2. Using a layered approach that included molecular docking with biological experiments, the authors described calpeptin (**34**) as a potent and specific inhibitor of SARS-CoV-2. Compound **34** inhibits the surface protein by binding with RBD with a high affinity. The location of the ligand in the binding interface of RBD–ACE2 is characterized by the formation of hydrogen bridges with S_1_494 and Y_1_453 and π–π and π–cation stacking interactions with Y_1_505 and R_1_403 (Figure 12B).

All these amino acid residues are important in the interaction of RBD with ACE2 [96]. Mutation N501Y, which occurs in all virulent strains of the virus, somewhat weakens the binding of agent **34** to RBD. However, in any case, substance **34** inhibits various strains of coronavirus and the energy parameters of binding the ligand to the domain correlate with the results of biological experiments [95].

In [30], in silico screening of 2467 naturally occurring compounds allowed authors to select five presumably active compounds against SARS-CoV-2, including H69C2 (**35**) (Figure 12C). Based on the data of native mass spectroscopy and surface plasmonic resonance, the authors showed that substance **35** can bind to the RBD with a dissociation constant K_D_ = 0.0947 μM. According to the results of the molecular docking procedure, molecule **35** is located in the binding interface of the domain and the cell enzyme to form hydrogen bonds with key amino acids of the domain, namely E_1_493 and S_1_494, and with R_1_403 and D_1_405. Hydrophobic interactions with L_1_455, Y_1_495, and Y_1_505 were also observed. In addition, the authors of [30] estimated the affinity of agent **35** to the RBD of mutant strains. Four key RBD mutations (K417N, L452R, E484K/Q, and N501Y), were considered in calculations and it has been shown that amino acids at position 452 and 484 do not contact molecule **35** and therefore do not affect ligand binding. The other two mutations, by contrast, result in an increase in the affinity of **35** to the binding site. As a result, the authors propose to consider this compound as a possible scaffold structure. In view of its potential hepatotoxicity, structural modification of this compound is recommended.

The practice of drug repurposing has enabled the authors of [97,98] to suggest that the cardiac glycoside digitoxin (**36**) may also inhibit the binding of RBD with ACE2. The compound was reported to be active against DNA and RNA viruses such as cytomegalovirus, herpes simplex virus, influenza virus, and coronavirus [97]. The IC_50_ values, characterizing the activity of glycoside **36** against SARS-CoV-2, range from 0.1 to 0.2 μM [99]. Based on the results of molecular modeling (molecular docking procedures [97,98] and molecular dynamic simulations [97]) it is assumed that agent **36** binds with the RBD to form hydrogen bonds with amino acids that are part of the motif of binding the domain to the enzyme, namely Y_1_453, V_1_417, and G_1_485. A steroid fragment of molecule **36** is embedded in the hydrophobic pocket formed by the residues of Y_1_489, E_1_484, G_1_485, and F_1_486. Despite the significant structural differences in the compounds (Figure 12), the authors of [30,87,91,95,97,98] suggest that molecules (**32**–**36**) bind with the interface of RBD–ACE2 to form intermolecular interactions with the same amino acids.

**Figure 13:**
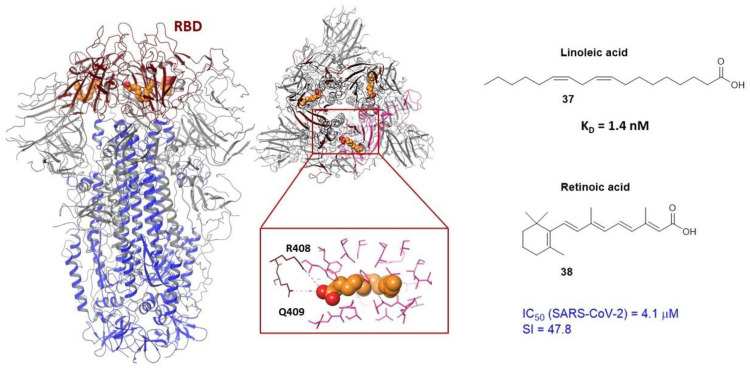
The location of the linoleic acid in the hydrophobic pocket and the anchor amino acids of the RBD of the neighboring protomer are shown in dark red.

The protein database [13] presents the geometric parameters of the S-protein of the SARS-CoV-2 in complex with linoleic acid (**37**) Figure 13 [100]. Linoleic acid (LA) itself does not show any antiviral properties but enhances the effect of remdesivir. The results of biological tests on live SARS-CoV-2 viral material show synergies: the dose of remdesivir needed to suppress viral replication was markedly reduced with the addition of fatty acid. LA binds in the S_1_ subunit of the surface protein (Figure 13) in the hydrophobic binding pocket located in the RBD. The molecule is located in such a way that its hydrophobic part forms hydrophobic interactions with surrounding hydrophobic amino acids and the carboxyl group contacts R_1_408 and Q_1_409 of the neighboring RBD. The basic principle of the LA effect is to stabilize the closed conformation of the surface protein.

In addition, the results of molecular dynamic simulations of it in the open protein conformation demonstrate high affinity of the molecule to the binding site within 500 ns. It should be noted here that the hydrophobic pocket is removed from the motif of binding RBD to ACE2. However, according to the authors of [100], a similar binding pocket is present in most pathogenic coronaviruses as there is a relationship between viral infection and fatty acid metabolism. In addition, the amino acid residues of SARS-CoV-1 and SARS-CoV-2 show conservatism, including anchor residues R_1_408 and E_1_409. The authors propose to develop entry inhibitors using this hydrophobic binding pocket.

Finally, we can note retinoic acid (**38**) which inhibits SARS-CoV-2 in micromolar concentrations (Figure 13). It is remarkable that the authors [100] recorded the geometric parameters of complex retinoic acid with an S-protein by cryo-electron microscopy. The data corresponds to PDB code 7Y42. As of the end of January 2023, these are so far the only parameters of ligands in the protein that can be used to develop new inhibitors of the S-protein that bind to RBD.

### 3.4. Binding Site of the N-Terminal Domain of S_1_

**Figure 14:**
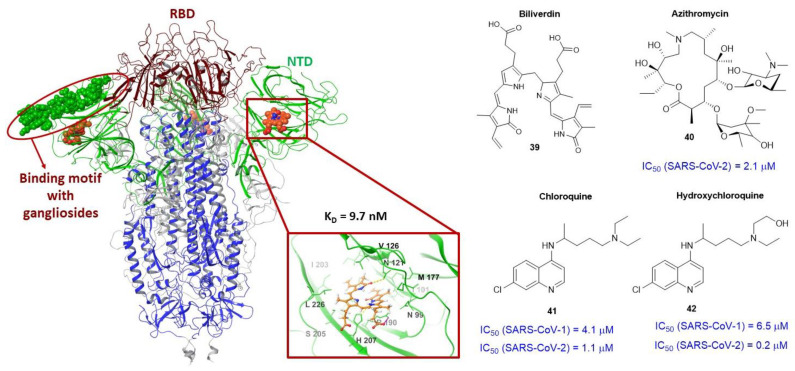
Structures binding in the NTD domain of S-protein. Biliverdin is shown in orange and hydrogen and salt bridges are shown in yellow and purple dotted lines.

The N-terminal domain of the S-protein of coronaviruses contains approximately 290 amino acid residues. Based on data from cryo-electron microscopy and X-ray crystallography, the authors of [101] determined the binding pocket of biliverdin (**39**), an intermediate product of hemoglobin metabolism. The binding site is located (Figure 14) in the NTD of the surface protein. According to the authors, the metabolite fits snugly into the binding pocket, which is saturated with hydrophobic amino acid residues. A series of intermolecular interactions are formed between the atoms of biliverdin and the residues of N_1_121, R_1_190, and H_1_207 (Figure 14). The binding of metabolite **39** to the S-protein is characterized by a dissociation constant K_D_ = 9.8 ± 1.3 nM. According to [101], biliverdin weakens the reactivity of the S-protein of SARS-CoV-2 in physiological concentrations. In addition, elevated bilirubin levels are correlated with mortality symptoms among COVID-19 patients. These studies are interesting in terms of understanding the immune response and may possibly be useful for the development of small molecule inhibitors.

The function of the N-terminal domain of the surface protein is not well understood [101]. It is known [102] that NTD-segment 111 to 158 amino acid is the motive for ganglioside binding (Figure 14). In other words, it is the site of attachment of the virus to lipid rafts: a section of plasma membrane cells enriched with glycosphingolipids and cholesterol. It is supposed that the interaction of this zone with lipid rafts subsequently helps the contact of RSD with cellular ACE2. Theoretically, the ganglioside-binding domain of the spike protein can be considered as a potential binding site for entry inhibitors [102].

The broad-spectrum antibiotic azithromycin (**40**) exhibits antiviral activity [99] against SARS-CoV-2 at micromolar concentrations (Figure 14). According to molecular simulation data, azithromycin [103,104] can bind to conserved amino acid residues of the ganglioside-binding domain NTD, namely with E_1_143, F_1_135, and N_1_137. In addition, ref. [104] shows that agent **40** can bind to M^PRO^ with high affinity. The conclusion is made on the basis of molecular docking data.

In addition to azithromycin (**40**), two other compounds chloroquine (**41**) and hydroxychloroquine (**42**) are active against SARS-CoV-1 [104,105] and SARS-CoV-2 [99] (Figure 14). In addition, it is mentioned in [104] that treatment with chloroquine and its analog **42** effectively reduces the viral load in patients with COVID-19. It is supposed that the likely mechanism of antiviral action of chloroquine (**41**) and hydroxychloroquine (**42**) is to inhibit the main protease. However, full-scale molecular modeling allowed the authors [102] to suggest that in the presence of **41** and **42**, the viral S-protein loses its ability to attach to lipid rafts. At the same time, the main mechanism of action of **41** and **42** is their competing interaction with gangliosides, and not with the surface protein itself. Despite the described results on the activity of **41** and **42** against SARS-CoV-2, in clinical practice their activity was low, and both drugs are now excluded from the recommendations for the treatment of COVID-19.

### 3.5. Binding Sites of the S_2_

**Figure 15:**
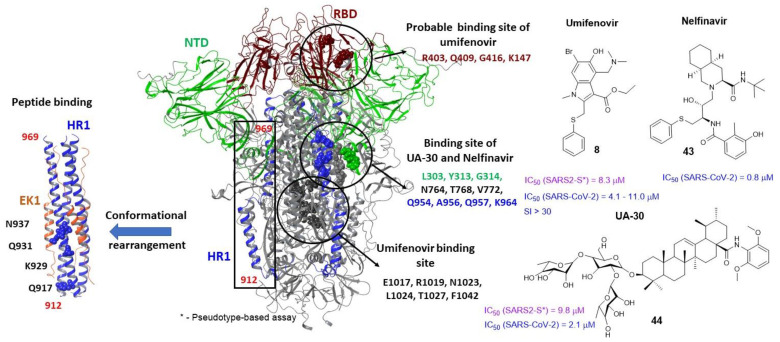
Binding sites of a number of S_2_-protein fusion inhibitors. Key amino acids are indicated.

From an evolutionary point of view, the RBD of coronaviruses is the most mutable area, which greatly complicates the development of broad-spectrum antiviral drugs. The second (S_2_) subunit of the glycoprotein of the coronavirus is a much more conservative part of the protein [12]. The region of heptad repeats 1 (HR1) of the S_2_ forms a homotrimeric complex that, as a result of conformational rearrangements, releases three highly conserved hydrophobic furrows on the surface associated with heptad repeat 2 (HR2). A structure composed of six spirals is formed during the fusion process and helps to bring the viral and cell membranes closer together to form a fusion pore [77]. Peptides derived from the HR2 region of type I fusion proteins, such as HIV-1, respiratory syncytial virus, Ebola virus, and a number of others can compete with viral heptad repeat 1 and effectively inhibit viral infection [83,106]. Peptide binding occurs after conformational rearrangements, when HR1 forms elongated α-helices (Figure 15). In conformational rearrangements, HR1 are in a compressed state until activation (lowering the pH). Stabilization of the “compressed” or pre-fusion state by small molecules can prevent conformational transitions and, as a consequence, inhibit the fusion of viral and cell membranes. However, the binding site of small inhibitors in the S_2_ spike protein of the coronavirus is still a controversial issue.

Umifenovir (**8**), an inhibitor of the influenza virus [45], was considered one of the first candidates as a potential inhibitor of the second subunit of the S-protein. Umifenovir is moderately active against SARS-CoV-2 virus in in vitro tests at concentrations between 4.1 and 11.0 μM (Figure 15) [107,108]. Based on molecular modeling data [109], a possible binding site **8** in the region of heptad repeats is described (Figure 15). As described above, Umifenovir binds in the stem region of the influenza virus hemagglutinin, preventing the transition from pre- to post-fusion conformation and, as a consequence, fusion of the viral and cell membranes. Influenza hemagglutinin and the S-spike protein of the SARS-CoV-2 coronavirus are type I surface proteins with a similar fusion mechanism. The presence of similar heptad repeats in these proteins suggests similar hydrophobic cavities in the space between the α-helices of the stem part of the protein. Based on the analysis of protein sequences of subunits HA_2_ and S_2_, the author of [109] determined the potential binding site of Arbidol in a small region S_2_ of the coronavirus domain. It is assumed that **8** interacts with key amino acid residues of the stem and effectively prevents the transition from pre- to post-fusion conformation. Interestingly, the authors of [110] describe an alternative binding site **8** located in the RBD at the zone of contact with the ACE2 (Figure 15). In the open access, the publication [110] appeared earlier than [109], but for a long time, it was in the pre-viewer stage. Why the authors of [110] chose the RBD as the binding site **8** is not clear. However, these two conflicting works inspired the authors of [111] to carry out large-scale molecular modeling, including a multiligand approach, and estimate the energy profile of potential binding sites of Umifenovir. Based on the results of molecular dynamic simulations, it was shown that **8** binds exactly in the stem part of the domain. The S-protein complex with three molecules of the ligand bound in the heptad repeats of the protein remains stable during the 300 ns simulation.

The HIV-1 protease inhibitor nelfinavir (**43**) also inhibits protease 3CL of SARS-CoV-1 [99]. In addition, it is active against SARS-CoV-2. It is very logical to assume that nelfinavir can inhibit the main protease M^PRO^ SARS-CoV-2. In [112,113], it is shown using molecular modeling that compound **43** demonstrates a pronounced affinity to the active site M^PRO^. In addition, nelfinavir inhibits fusion of viral and cell membranes and may be considered as an inhibitor of SARS-CoV-2 entry (Figure 15). The results of the molecular docking allowed the authors of [114] to determine a possible binding region **43** located between the helixes of HR1 and the lower part of the N-terminal domain (Figure 15). A similar binding site of fusion inhibitors is also described in [115]. The compound UA-30 (**44**) inhibits the surface protein SARS-CoV-2, binds in the cavity between two subunits, and thereby stabilizes the pre-fusion conformation of the protein. The binding site is determined on the basis of molecular modeling and data from biological experiments to create a resistant strain of the virus.

**Figure 16:**
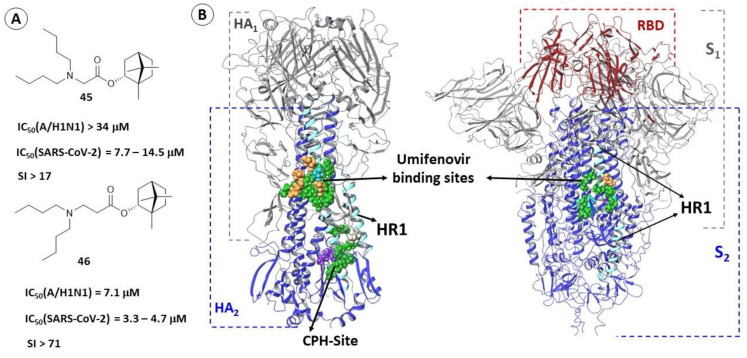
Derivatives of (-)-borneol ester are inhibitors of HA and S-protein. Structures of derivatives of (-)-borneol esters (**A**): the ranked value of IC_50_ depends on the inhibitory ability against different strains of the virus (adapted from [116]). A comparison (**B**) of the pharmacophoric profile of the binding sites of the stem part of HA_2_ and S_2_: the secondary structure of the heptad repeat (HR1) (a. a. 910–985) in one protein protomer is shown in blue. Hydrophobic amino acids are shown in green and positively and negatively charged amino acids are in purple and light orange, respectively. Polar amino acids are shown in blue.

Biological experiments on the SARS-CoV-2 virus of three strains and application of a pseudoviral system allowed [116] to identify two active compounds (**45** and **46**) among a number of Borneol ester derivatives (Figure 16A). These compounds inhibit the surface viral spike protein. Molecular modeling methods (molecular docking procedures and a number of molecular dynamic simulations) suggested that inhibitors **45** and **46** bind in a cavity located in the second subunit of the S-protein in the region of central heptad repeats (Figure 13).

The binding site of these compounds was selected on the basis of the following inferences: firstly, compounds **45** and **46** exhibit similar activity against SARS-CoV-2 of Wuhan, Delta, and Omicron strains. The second subunit is the least variable part of the S-protein, and only isolated mutations have been found in the region of heptad repeats [12]. Secondly, the borneol esters are active against influenza virus [117] and compounds with a similar scaffold (**15,** Figure 6) inhibit the fusogenic activity of HA and bind in the hydrophobic cavities of the stem part of the domain, namely in the TBHQ site and CPH site [52]. The alignment of amino acid sequences and the comparison of pharmacophoric profiles of probable binding sites allowed the authors of the work to describe the mechanism of inhibitory action of these molecules. Derivatives of (-)-borneol esters (**45** and **46**) can bind in hydrophobic spaces between α-helixes of the glycoprotein subunit S_2_ (Figure 16B), thereby inhibiting the fusogenic activity of the protein.

The continuing coronavirus pandemic since 2020 has shown the need to develop new antiviral drugs. The surface viral protein SARS-CoV-2 is the second most popular target. Results of molecular modeling methods are often given without any experimental evaluation. The practice of drug repurposing is most often reduced to in silico screening of a library of FDA-approved compounds. Only in rare cases does science use a set of theoretical and experimental methods that confirm the activity of a substance against the surface protein of the coronavirus. The use of molecular modeling methods is more justified than ever. However, the lack of geometric parameters of the S-protein–ligand complex greatly complicates the search for inhibitors and understanding of the mechanism of their antiviral activity.

## 4. Conclusions

In this review, we described inhibitors of influenza virus and coronavirus fusion proteins. Of course, these viruses belong to different families. The similarity lies only in the mechanism of fusion of the viral and cell membranes, the key role of which is played by the surface proteins hemagglutinin and spike protein. However, the choice of these two proteins is not accidental. Firstly, we describe two almost borderline cases. The HA protein is well described. The mechanism of its action is clear. The protein data base contains the geometric parameters of the protein with ligands in various binding sites. The spike protein was also studied and its mechanism of action was described, but with some contradictions. However, during two years of the ongoing pandemic, there are still no data on geometric parameters of the protein in combination with small molecular weight ligands. For this reason, it is extremely difficult to describe the mechanism of antiviral action of compounds. Secondly, despite the structural differences of proteins, some cavities considered as potential inhibitor binding sites have a similar pharmacophoric profile. This explains the antiviral activity of Umifenovir and borneol esters against the influenza virus and the S-protein of coronavirus. In addition, this fact may be a loophole for the search and development of broad-spectrum antiviral drugs.

Molecular modeling (docking or molecular dynamic simulations) and quantum-chemical calculations are powerful tools that, with the right approach, can greatly help the task of finding and developing antiviral drugs. However, in order to find the binding site of the entry inhibitors and to create an adequate model for theoretical calculations, data from biological experiments are needed, at a minimum to confirm the choice of a potential biological target, such as an experiment on the time of addition. Obtaining resistant strains of the virus followed by sequencing and localization of amino acid substitutions and NMR studies suggest a likely binding site. As a rule, after that molecular modeling methods are used to visualize and describe the mechanism of antiviral activity of the studied compounds, including the selective one. A clear understanding of the mechanism of fusion, indicating which specific amino acid residues are involved in conformational rearrangements, can help researchers to determine the binding sites of potential entry inhibitors. We hope that the results of scientific publications described in this review will be useful to researchers for finding a “magic” molecule and creating a non-toxic antiviral drug with a broad spectrum of activity.

## Data Availability

Not applicable.

## Supplementary Materials

The following supporting information can be downloaded at: https://www.mdpi.com/article/10.3390/v15040902/s1. Table S1: PDB codes of complexes HA of influenza virus and S-protein of coronavirus with ligands and Table S2: Inhibitors of the surface protein of influenza virus and SARS-CoV-2. References [17,28,29,30,32,33,34,37,43,44,45,46,47,48,49,50,51,52,53,57,59,60,61,62,72,73,74,75,86,87,89,91,92,95,97,98,99,100,101,102,103,104,105,106,109,110,111,112,113,114,115,116,118,119] were cited in the Supplementary Materials.

## Abbreviation

a. a.	amino acids
ACE2	angiotensin-converting enzyme 2
CoV	coronavirus
CTD	C-terminal domain
CPH	camphecene
F	fusion protein
FP	fusion peptide
G	glycoprotein
HA	influenza virus hemagglutinin
HA_1_	first subunits of influenza virus hemagglutinin (global head)
HA_2_	second subunits of influenza virus hemagglutinin (stem part)
HIV-1	Human immunodeficiency virus
HR	heptad repeat
HTS	High-throughput screening
LA	linoleic acid
MERS-CoV	middle east respiratory syndrome
RBD	receptor binding domain of coronavirus
RBS	hemagglutinin receptor binding site
NTD	N-terminal domain
PIV	parainfluenza virus
QM	quantum mechanic
S	S-protein or glycoprotein of coronavirus
S_1_	first subunit of coronavirus S-protein
S_2_	second subunit of coronavirus S-protein
SI	selectivity index
SARS	severe acute respiratory syndrome
TBEV	tick-borne encephalitis virus
TMD	trans-membrane domain
VSV	vesicular stomatitis virus
