# Supplementary material for: Molecular Modeling of Viral Type I Fusion Proteins: Inhibitors of Influenza Virus Hemagglutinin and the Spike Protein of Coronavirus
Source: Viruses. 2023 Mar 31;15(4):902. doi: 10.3390/v15040902 (PMC10142020; doi:10.3390/v15040902)
Supplement: Supplementary file 1 [file viruses-15-00902-s001.zip › viruses-2314544-supplementary.docx]

**Table S1.** PDB codes of complexes HA of influenza virus and S-protein of coronavirus with ligands**.**

| PBD ID | Virus strain | Ligand | Binding site | Reference |
| --- | --- | --- | --- | --- |
| Haemagglutinin | | | | |
| 6CF5 | A/Viet Nam/1203/2004(H5N1) | 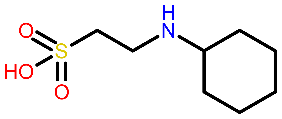  N-cyclohexyltaurine | Receptor binding site of HA_1_ | [1] |
| 6CEX | A/Hong Kong/1/1968 (H3N2) |  | Receptor binding site of HA_1_ |  |
|  |  |  | Stem part of НА_2_, hydrophobic cavity between the short and long α-helices of two protomers of HA_2_ |  |
| 3EYM | A/Aichi/2/1968 H3N2 | 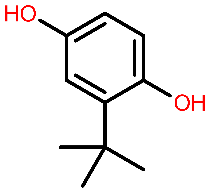TBHQ | Stem part of НА_2_, hydrophobic cavity between the short and long α-helices of two protomers of HA_2_ | [2] |
| 3EYK | A/Mallard/Astrakhan/244/1982 H14N6 |  |  |  |
| 5T6N | A/H3N2 | 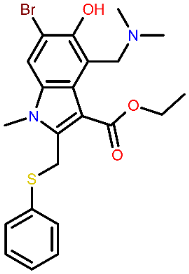  Umifenovir (Arbidol) | Stem part of НА_2_, hydrophobic cavity between the short and long α-helices of two protomers of HA_2_ | [3] |
| 5T6S | A/H7N9 |  |  |  |
| 5W6U | A/Puerto Rico/8/1934 (H1N1) | Cyclic peptide P2 | Highly conserved epitope of the stem part of the domain | [4] |
| 5W6I |  | Cyclic peptide P3 |  |  |
| 5W5U |  | Cyclic peptide P4 |  |  |
| 5W5S |  | Cyclic peptide P5 |  |  |
| 5W6R |  | Cyclic peptide P6 |  |  |
| 5W6T |  | Cyclic peptide P7 |  |  |
| 6WCR | A/Puerto Rico/8/1934 (H1N1) | 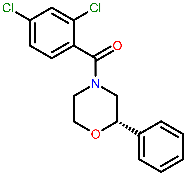F0045(S) | Highly conserved epitope of the stem part of the domain | [5] |
| 6CF7 | A/Solomon Islands/3/2006(H1N1) | 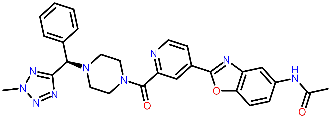  JNJ4796 | Highly conserved epitope of the stem part of the domain | [6] |
| 6CFG | A/Vietnam/1203/2004 (H5N1) |  |  |  |
| 6VMZ | A/Chicken/Vietnam/30/2003(H5N1) | 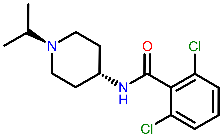CBS1117 | Highly conserved epitope of the stem part of the domain | [7] |
| S-protein | | | | |
| 6ZB4 | SARS-CoV-2 (WT, Wuhan) | 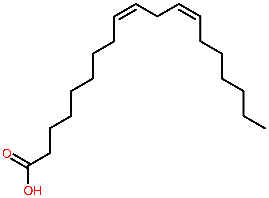  Linolec acid | Hydrophobic pocket in RBD of S_1_ | [8] |
| 7OD3 | SARS-CoV-2 (Alfa B.1.1.7, UK) |  |  |  |
| 7Y42 | SARS-CoV-2 (WT, Wuhan) |   Retinoid acid | RBD | [9] |
| 7B62 | SARS-CoV-2 (WT, Wuhan) | 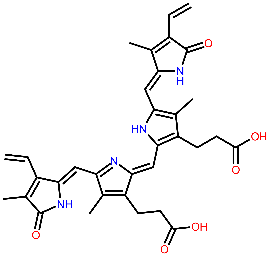  Biliverdin | N—terminal domain of S_1_ SARS-CoV-2 (amino acids 14 to 330) | [10] |
| 7NT9 |  |  | N—Terminal Domain S_1_ SARS-CoV-2 (amino acids 14 to 1139) |  |
| 7C53 | SARS-CoV-2 (WT, Wuhan) | Peptide EK1 | HR1 | [11] |
| 5ZVK | MERS-CoV | Peptide EK1 | HR1 | [12] |

**Table S2.** Inhibitors of surface protein of influenza virus and SARS-CoV-2.

| **№** | **Structure** | **IC_50_, μM** | **SI** | **K_D_, μM** | **Binding site** | **Reference** |
| --- | --- | --- | --- | --- | --- | --- |
| **Influenza virus hemagglutinin** | | | | | | |
| 1 |   **SA-α-2,3-Gal** | n/d | n/d | 3-5×10^3^ | HA_1_: RBS | [1,13] |
| 2 |   **SA-α-2,6-Gal** | n/d | n/d | 3-5×10^3^ | HA_1_: RBS | [1,13] |
| 3 |  | IC_50_(A/H1N1) = 5.5  IC_50_(A/H3N2) = 10.4 | > 8  > 9 | n/d | HA_1_: RBS | [14] |
| 4 |   **Aureonitol** | IC_50_(A/H1N1) = 0.4  IC_50_(A/H3N2) = 0.3 | 3420  4571 | n/d | HA_1_: RBS | [15] |
| 5 |   **N-cyclohexyltaurine** | n/d | n/d | > 20×10^3^ | HA_1_: RBS  НА_2_: stem part – the site of the fusion peptide  (TBHQ-site) | [1] |
| 6 |   **NSC85561** | IC_50_ (A/H1N1) = 2.4 | 289 | n/d | HA_1_: RBS | [16] |
| 7 |   **TBHQ** | IC_50_(A/H3N2) ~ 6.0 | > 8 | n/d | НА_2_: stem part – the site of the fusion peptide  (TBHQ-site) | [2] |
| 8 |   **Umifenovir (Arbidol)** | IC_50_ (A/H1N1) = 26.2  IC_50_ (A/H3N2) = 6.2  IC_50_ (A/H5N1) = 15.7 | 20  > 4  > 8 | 5.6 – 7.9 (H1)  18.8 – 44.3 (H3) | НА_2_: stem part – the site of the fusion peptide  (TBHQ-site) | [3,17,18] |
| 9 |   **Compound 11** | n/d | n/d | 550 ×10^3^ (H1)  100×10^3^ (H3) | НА_2_: stem part – the site of the fusion peptide  (TBHQ-site) | [18] |
| 10 |  | IC_50_ (A/H3N2) = 7.5 | > 13 | n/d | НА_2_: stem part – the site of the fusion peptide  (TBHQ-site) | [19] |
| 11 |  | IC_50_ (A/H1N1) = 19.0 | 45 | n/d | НА_2_: stem part – the site of the fusion peptide  (TBHQ-site) | [20] |
| 12 |  | IC_50_ (A/H1N1) = 7.5 | 189 | n/d | НА_2_: stem part – the site of the fusion peptide  (TBHQ-site) | [21] |
| 13 |  | IC_50_ (A/H1N1) = 6.0 | 231 | n/d | НА_2_: stem part – the site of the fusion peptide  (TBHQ-site) | [22] |
| 14 |   **Camphecene** | IC_50_ (A/H1N1) = 1.1  IC_50_ (A/H3N2) = 10.3  IC_50_ (A/H5N1) = 8.0 | 661  75  97 | н/д | НА_2_: stem part – the site of the fusion peptide  (TBHQ-site); proteolysis site (CPH-site) | [23–26] |
| 15 |  | IC_50_ (A/H1N1) = 2.4 | 546 | n/d | НА_2_: stem part – the site of the fusion peptide  (TBHQ-site); proteolysis site (CPH-site) | [27] |
| 16 |  | IC_50_ (A/H1N1) = 1.2×10^-3^ | 1250 | n/d | НА_2_: stem part – the site of the fusion peptide  (TBHQ-site) | [28] |
| 17 |   **MBX2329** | IC_50_ (A/H1N1) = 0.4  IC_50_ (A/H5N1) = 5.9 | > 222  > 17 | n/d | HA_2_: stem part – epitope | [29,30] |
| 18 |   **MBX2346** | IC_50_ (A/H1N1) = 0.3  IC_50_ (A/H5N1) = 3.6 | > 333  > 27 | n/d | НА_2_: stem part – epitope | [29] |
| 19 |  | IC_50_ (A/H1N1) = 4.6 | > 22 | n/d | НА_2_: stem part – the site of the fusion peptide  (TBHQ-site)  possibly an epitope | [31] |
| 20 |  | IC_50_ (A/H1N1) = 8.4 | 6 | n/d | НА_2_: stem part – the site of the fusion peptide  (TBHQ-site)  possibly an epitope | [31] |
| 21 |   **JNJ4796** | IC_50_ (A/H1N1) = 0.02 | 6500 | n/d | НА_2_: stem part – epitope | [6] |
| 22 | **F0045(S)** | IC_50_ (A/H1N1) = 8.8 | > 14 | n/d | НА_2_: stem part – epitope | [5] |
| 23 | **F0045®** | IC_50_ (A/H1N1) = 43.0 | > 3 | n/d | НА_2_: stem part – epitope | [5] |
| 24 | ****  **CBS1117** | IC_50_ (A/H1N1) = 0.07 | 3914 | n/d | НА_2_: stem part – epitope | [7] |
| 25 |  | IC_50_ (A/H1N1) = 0.03 | > 6666 | n/d | НА_2_: stem part – epitope | [32] |
| 26 | ****  **Tanshinone IIA** | IC_50_ (A/H1N1) = 0.03 | 1236 | n/d | НА_2_: stem part – epitope | [33] |
| 27 |   **Stachyflin** | IC_50_ (A/H1N1) = 0.05  IC_50_ (A/H3N2) > 6.5  IC_50_ (A/H5N1) = 1.9 | n/d | n/d | НА_2_: stem part – cavity between two α-helixes | [34] |
| 28 |   **Ginsamid** | IC_50_ (A/H1N1) = 0.15 | 7500 | n/d | НА_2_: stem part – the place of proteolysis (CPH-site) | [35] |
| 29 |  | IC_50_ (A/H1N1) = 1.9 | 26 | n/d | НА_2_: stem part – the place of proteolysis (CPH-site) | [36] |
| 30 |   **IY7640** | IC_50_ (A/H1N1) = 0.7  IC_50_ (A/H3N2) = 83.1  IC_50_ (A/H5N1) = 59.6 | 1143  10  13 | n/d | НА_2_: stem part – the cavity between the TBH-site and the epitope | [37] |
| 31 |   **M090** | IC_50_ (A/H1N1) = 2.2 | 11 | n/d | HA_2_: cavity at the interface of two subunits between a long α-helix and a loop connecting two α-helixes | [38] |
| **Spike protein of coronavirus** | | | | | | |
| 32 |   **SSAA09E2** | IC_50_ (SARS1-S) = 9.7 | > 20 | n/d | S_1_: interface of binding RBD with ACE-2 | [39,40] |
| 33 |   **Nilotinib** | IC_50_ (SARS-CoV-2) = 1.9 | 16 | n/d | S_1_: interface of binding RBD with ACE-2 | [40–42] |
|  |  |  |  |  | Active site of M^PRO^  NSP12-NSP7-NSP8 | [43]  [44] |
| 34 |   **Calpeptin** | IC_50_ (SARS1-S) = 0.8  IC_50_ (SARS2-S) = 0.1  IC_50_ (SARS-CoV-2) = 3.8 | > 230  > 1612  > 50 | n/d | S_1_: interface of binding RBD with ACE-2 | [45] |
| 35 |   **N69C2** | IC_50_ (SARS-CoV-2) = 85.7 | 3 | 0.09 | S_1_: interface of binding RBD with ACE-2 | [46] |
| 36 |   **Digitoxin** | IC_50_ (SARS-CoV-2) = 0.1 | n/d | n/d | S_1_: interface of binding RBD with ACE-2 | [42,47,48] |
| 37 |   **Linolec acid** | n/d | n/d | 1.4×10^3^ | S_1_: hydrophobic pocket of RSD | [8] |
| 38 |   **Retinoid acid** | IC_50_ (SARS-CoV-2) = 4.1 | 47.8 | n/d | S_1_: interface of binding RBD with ACE-2 | [9] |
| 38 |   **Biliverdin** | n/d | n/d | 9.7×10^3^ | S_1_: NTD | [10] |
| 39 |   **Azithromycin** | IC_50_ (SARS-CoV-2) = 2.1 | n/d | n/d | S_1_: NTD | [42,49,50] |
|  |  |  |  |  | Active site of M^PRO^ | [50] |
| 40 |   **Chloroquine** | IC_50_ (SARS-CoV-1) = 4.1  IC_50_ (SARS-CoV-2) = 1.1 | n/d | n/d | Gangliosides | [42,51,52] |
|  |  |  |  |  | S_1_: interface of binding RBD with ACE2  Active site of M^PRO^ | [50] |
| 41 |   **Hydroxychloroquine** | IC_50_ (SARS-CoV-1) = 6.5  IC_50_ (SARS-CoV-2) = 0.2 | n/d | n/d | Gangliosides | [42,51,52] |
|  |  |  |  |  | S_1_: interface of binding RBD with ACE2  Active site of M^PRO^ | [50] |
| 8 |  **Umifenovir** | IC_50_ (SARS2-S) = 8.3  IC_50_ (SARS-CoV-2) = 4.1 | > 15  > 30 | n/d | S_1_: interface of binding RBD with ACE2 | [53] |
|  |  |  |  |  | S_2_: area of heptad repeat | [54,55] |
| 42 |   **Nelfinavir** | IC_50_ (SARS-CoV-2) = 0.8 | n/d | n/d | S_2_: cavity between the HR1 and the lower part of the N-terminal domain | [56] |
|  |  |  |  |  | Active site of M^PRO^ | [57,58] |
| 43 |   **UA-30** | IC_50_ (SARS2-S) = 9.8  IC_50_ (SARS-CoV-2) = 2.1 | n/d | 0.78 | S_2_: cavity between the HR1 and the lower part of the N-terminal domain | [59] |
| 44 |  | SARS-CoV-2  IC_50_ (Wuhan) = 9.6  IC_50_ (Delta) = 17.6  IC_50_ (Omicron) = 7.7 | 32  17  40 | n/d | S_2_: area of heptad repeat | [60] |
| 45 |  | SARS-CoV-2  IC_50_ (Wuhan) = 4.7  IC_50_ (Delta) = 3.5  IC_50_ (Omicron) = 3.3 | 71  96  102 | n/d | S_2_: area of heptad repeat | [60] |

References

1. Kadam, R.U.; Wilson, I.A. A small-molecule fragment that emulates binding of receptor and broadly neutralizing antibodies to influenza A hemagglutinin. *Proc. Natl. Acad. Sci.* **2018**, *115*, 4240–4245, doi:10.1073/pnas.1801999115.

2. Russell, R.J.; Kerry, P.S.; Stevens, D.J.; Steinhauer, D.A.; Martin, S.R.; Gamblin, S.J.; Skehel, J.J. Structure of influenza hemagglutinin in complex with an inhibitor of membrane fusion. *Proc. Natl. Acad. Sci.* **2008**, *105*, doi:10.1073/pnas.0807142105.

3. Kadam, R.U.; Wilson, I.A. Structural basis of influenza virus fusion inhibition by the antiviral drug Arbidol. *Proc. Natl. Acad. Sci. U. S. A.* **2017**, *114*, 206–214, doi:10.1073/pnas.1617020114.

4. Kadam, R.U.; Juraszek, J.; Brandenburg, B.; Buyck, C.; Schepens, W.B.G.; Kesteleyn, B.; Stoops, B.; Vreeken, R.J.; Vermond, J.; Goutier, W.; et al. Potent peptidic fusion inhibitors of influenza virus. *Science (80-. ).* **2017**, *358*, 496–502, doi:10.1126/science.aan0516.

5. Yao, Y.; Kadam, R.U.; Lee, C.C.D.; Woehl, J.L.; Wu, N.C.; Zhu, X.; Kitamura, S.; Wilson, I.A.; Wolan, D.W. An influenza A hemagglutinin small-molecule fusion inhibitor identified by a new high-throughput fluorescence polarization screen. *Proc. Natl. Acad. Sci. U. S. A.* **2020**, *117*, 18431–18438, doi:10.1073/pnas.2006893117.

6. van Dongen, M.J.P.; Kadam, R.U.; Juraszek, J.; Lawson, E.; Brandenburg, B.; Schmitz, F.; Schepens, W.B.G.; Stoops, B.; van Diepen, H.A.; Jongeneelen, M.; et al. A small-molecule fusion inhibitor of influenza virus is orally active in mice. *Science (80-. ).* **2019**, *363*, doi:10.1126/science.aar6221.

7. Antanasijevic, A.; Durst, M.A.; Cheng, H.; Gaisina, I.N.; Perez, J.T.; Manicassamy, B.; Rong, L.; Lavie, A.; Caffrey, M. Structure of avian influenza hemagglutinin in complex with a small molecule entry inhibitor. *Life Sci. Alliance* **2020**, *3*, doi:10.26508/LSA.202000724.

8. Toelzer, C.; Gupta, K.; Yadav, S.K.N.N.; Borucu, U.; Davidson, A.D.; Kavanagh Williamson, M.; Shoemark, D.K.; Garzoni, F.; Staufer, O.; Milligan, R.; et al. Free fatty acid binding pocket in the locked structure of SARS-CoV-2 spike protein. *Science (80-. ).* **2020**, *370*, 725–730, doi:10.1126/science.abd3255.

9. Tong, L.; Wang, L.; Liao, S.; Xiao, X.; Qu, J.; Wu, C.; Zhu, Y.; Tai, W.; Huang, Y.; Wang, P.; et al. A Retinol Derivative Inhibits SARS-CoV-2 Infection by Interrupting Spike-Mediated Cellular Entry. *MBio* **2022**, *13*, doi:10.1128/mbio.01485-22.

10. Rosa, A.; Pye, V.E.; Graham, C.; Muir, L.; Seow, J.; Ng, K.W.; Cook, N.J.; Rees-Spear, C.; Parker, E.; dos Santos, M.S.; et al. SARS-CoV-2 can recruit a heme metabolite to evade antibody immunity. *Sci. Adv.* **2021**, *7*, doi:10.1126/sciadv.abg7607.

11. Xia, S.; Lan, Q.; Zhu, Y.; Wang, C.; Xu, W.; Li, Y.; Wang, L.; Jiao, F.; Zhou, J.; Hua, C.; et al. Structural and functional basis for pan-CoV fusion inhibitors against SARS-CoV-2 and its variants with preclinical evaluation. *Signal Transduct. Target. Ther.* **2021**, *6*, 288, doi:10.1038/s41392-021-00712-2.

12. Xia, S.; Yan, L.; Xu, W.; Agrawal, A.S.; Algaissi, A.; Tseng, C.-T.K.; Wang, Q.; Du, L.; Tan, W.; Wilson, I.A.; et al. A pan-coronavirus fusion inhibitor targeting the HR1 domain of human coronavirus spike. *Sci. Adv.* **2019**, *5*, doi:10.1126/sciadv.aav4580.

13. Li, M.; Wang, B. Computational studies of H5N1 hemagglutinin binding with SA-α-2, 3-Gal and SA-α-2, 6-Gal. *Biochem. Biophys. Res. Commun.* **2006**, *347*, 662–668, doi:10.1016/j.bbrc.2006.06.179.

14. Meng, L.; Su, Y.; Yang, F.; Xiao, S.; Yin, Z.; Liu, J.; Zhong, J.; Zhou, D.; Yu, F. Design, synthesis and biological evaluation of amino acids-oleanolic acid conjugates as influenza virus inhibitors. *Bioorganic Med. Chem.* **2019**, *27*, 115147, doi:10.1016/j.bmc.2019.115147.

15. Sacramento, C.Q.; Marttorelli, A.; Fintelman-Rodrigues, N.; De Freitas, C.S.; De Melo, G.R.; Rocha, M.E.N.; Kaiser, C.R.; Rodrigues, K.F.; Da Costa, G.L.; Alves, C.M.; et al. Aureonitol, a fungi-derived tetrahydrofuran, inhibits influenza replication by targeting its surface glycoprotein hemagglutinin. *PLoS One* **2015**, *10*, doi:10.1371/journal.pone.0139236.

16. Chang, Y.-J.; Yeh, C.-Y.; Cheng, J.-C.; Huang, Y.-Q.; Hsu, K.-C.; Lin, Y.-F.; Lu, C.-H. Potent sialic acid inhibitors that target influenza A virus hemagglutinin. *Sci. Rep.* **2021**, *11*, 8637, doi:10.1038/s41598-021-87845-0.

17. Boriskin, Y.; Leneva, I.; Pecheur, E.-I.; Polyak, S. Arbidol: A Broad-Spectrum Antiviral Compound that Blocks Viral Fusion. *Curr. Med. Chem.* **2008**, *15*, 997–1005, doi:10.2174/092986708784049658.

18. Wright, Z.V.F.; Wu, N.C.; Kadam, R.U.; Wilson, I.A.; Wolan, D.W. Structure-based optimization and synthesis of antiviral drug Arbidol analogues with significantly improved affinity to influenza hemagglutinin. *Bioorganic Med. Chem. Lett.* **2017**, *27*, 3744–3748, doi:10.1016/j.bmcl.2017.06.074.

19. Vanderlinden, E.; Göktaş, F.; Cesur, Z.; Froeyen, M.; Reed, M.L.; Russell, C.J.; Cesur, N.; Naesens, L. Novel Inhibitors of Influenza Virus Fusion: Structure-Activity Relationship and Interaction with the Viral Hemagglutinin. *J. Virol.* **2010**, *84*, 4277–4288, doi:10.1128/jvi.02325-09.

20. Ilyina, I. V.; Patrusheva, O.S.; Zarubaev, V. V.; Misiurina, M.A.; Slita, A. V.; Esaulkova, I.L.; Korchagina, D. V.; Gatilov, Y. V.; Borisevich, S.S.; Volcho, K.P.; et al. Influenza antiviral activity of F- and OH-containing isopulegol-derived octahydro-2H-chromenes. *Bioorganic Med. Chem. Lett.* **2021**, *31*, 127677, doi:10.1016/j.bmcl.2020.127677.

21. Ilyina, I. V.; Zarubaev, V. V.; Lavrentieva, I.N.; Shtro, A.A.; Esaulkova, I.L.; Korchagina, D. V.; Borisevich, S.S.; Volcho, K.P.; Salakhutdinov, N.F. Highly potent activity of isopulegol-derived substituted octahydro-2H-chromen-4-ols against influenza A and B viruses. *Bioorganic Med. Chem. Lett.* **2018**, *28*, 2061–2067, doi:10.1016/j.bmcl.2018.04.057.

22. Chernyshov, V. V.; Yarovaya, O.I.; Esaulkova, I.L.; Sinegubova, E.; Borisevich, S.S.; Popadyuk, I.I.; Zarubaev, V. V.; Salakhutdinov, N.F. Novel O-acylated amidoximes and substituted 1,2,4-oxadiazoles synthesised from (+)-ketopinic acid possessing potent virus-inhibiting activity against phylogenetically distinct influenza A viruses. *Bioorg. Med. Chem. Lett.* **2022**, *55*, 128465, doi:10.1016/j.bmcl.2021.128465.

23. Zarubaev, V.V.; Garshinina, A.V.; Tretiak, T.S.; Fedorova, V.A.; Shtro, A.A.; Sokolova, A.S.; Yarovaya, O.I.; Salakhutdinov, N.F. Broad range of inhibiting action of novel camphor-based compound with anti-hemagglutinin activity against influenza viruses in vitro and in vivo. *Antiviral Res.* **2015**, *120*, 126–133, doi:10.1016/j.antiviral.2015.06.004.

24. Sokolova, A.S.; Yarovaya, O.I.; Shernyukov, A. V.; Gatilov, Y. V.; Razumova, Y. V.; Zarubaev, V. V.; Tretiak, T.S.; Pokrovsky, A.G.; Kiselev, O.I.; Salakhutdinov, N.F. Discovery of a new class of antiviral compounds: Camphor imine derivatives. *Eur. J. Med. Chem.* **2015**, *105*, 263–273, doi:10.1016/j.ejmech.2015.10.010.

25. Zarubaev, V. V; Pushkina, E.A.; Borisevich, S.S.; Galochkina, A. V; Garshinina, A. V; Shtro, A.A.; Egorova, A.A.; Sokolova, A.S.; Khursan, S.L.; Yarovaya, O.I.; et al. Selection of influenza virus resistant to the novel camphor-based antiviral camphecene results in loss of pathogenicity. *Virology* **2018**, *524*, 69–77, doi:10.1016/j.virol.2018.08.011.

26. Borisevich, S.S.; Gureev, M.A.; Yarovaya, О.I.; Zarubaev, V. V.; Kostin, G.A.; Porozov, Y.B.; Salakhutdinov, N.F. Can molecular dynamics explain decreased pathogenicity in mutant camphecene-resistant influenza virus? *J. Biomol. Struct. Dyn.* **2021**, *40*, 5481–5492, doi:10.1080/07391102.2020.1871414.

27. Sokolova, A.S.; Yarovaya, O.I.; Baranova, D. V.; Galochkina, A. V.; Shtro, A.A.; Kireeva, M. V.; Borisevich, S.S.; Gatilov, Y. V.; Zarubaev, V. V.; Salakhutdinov, N.F. Quaternary ammonium salts based on (-)-borneol as effective inhibitors of influenza virus. *Arch. Virol.* **2021**, *166*, 1965–1976, doi:10.1007/s00705-021-05102-1.

28. Cihan-Üstündağ, G.; Zopun, M.; Vanderlinden, E.; Ozkirimli, E.; Persoons, L.; Çapan, G.; Naesens, L. Superior inhibition of influenza virus hemagglutinin-mediated fusion by indole-substituted spirothiazolidinones. *Bioorganic Med. Chem.* **2020**, *28*, 115130, doi:10.1016/j.bmc.2019.115130.

29. Basu, A.; Antanasijevic, A.; Wang, M.; Li, B.; Mills, D.M.; Ames, J.A.; Nash, P.J.; Williams, J.D.; Peet, N.P.; Moir, D.T.; et al. New Small Molecule Entry Inhibitors Targeting Hemagglutinin-Mediated Influenza A Virus Fusion. *J. Virol.* **2014**, *88*, 1447–1460, doi:10.1128/jvi.01225-13.

30. Guan, S.; Wang, T.; Kuai, Z.; Qian, M.; Tian, X.; Zhang, X.; Yu, Y.; Wang, S.; Zhang, H.; Li, H.; et al. Exploration of binding and inhibition mechanism of a small molecule inhibitor of influenza virus H1N1 hemagglutinin by molecular dynamics simulation. *Sci. Rep.* **2017**, *7*, doi:10.1038/s41598-017-03719-4.

31. Leiva, R.; Barniol-Xicota, M.; Codony, S.; Ginex, T.; Vanderlinden, E.; Montes, M.; Caffrey, M.; Luque, F.J.; Naesens, L.; Vázquez, S. Aniline-Based Inhibitors of Influenza H1N1 Virus Acting on Hemagglutinin-Mediated Fusion. *J. Med. Chem.* **2018**, *61*, 98–118, doi:10.1021/acs.jmedchem.7b00908.

32. Wang, A.; Li, Y.; Lv, K.; Gao, R.; Wang, A.; Yan, H.; Qin, X.; Xu, S.; Ma, C.; Jiang, J.; et al. Optimization and SAR research at the piperazine and phenyl rings of JNJ4796 as new anti-influenza A virus agents, part 1. *Eur. J. Med. Chem.* **2021**, *222*, 113591, doi:10.1016/j.ejmech.2021.113591.

33. Elebeedy, D.; Badawy, I.; Elmaaty, A.A.; Saleh, M.M.; Kandeil, A.; Ghanem, A.; Kutkat, O.; Alnajjar, R.; Abd El Maksoud, A.I.; Al-karmalawy, A.A. In vitro and computational insights revealing the potential inhibitory effect of Tanshinone IIA against influenza A virus. *Comput. Biol. Med.* **2022**, *141*, 105149, doi:10.1016/j.compbiomed.2021.105149.

34. Motohashi, Y.; Igarashi, M.; Okamatsu, M.; Noshi, T.; Sakoda, Y.; Yamamoto, N.; Ito, K.; Yoshida, R.; Kida, H. Antiviral activity of stachyflin on influenza A viruses of different hemagglutinin subtypes. *Virol. J.* **2013**, *10*, doi:10.1186/1743-422X-10-118.

35. Volobueva, A.S.; Yarovaya, O.I.; Kireeva, M. V.; Borisevich, S.S.; Kovaleva, K.S.; Mainagashev, I.Y.; Gatilov, Y. V.; Ilyina, M.G.; Zarubaev, V. V.; Salakhutdinov, N.F. Discovery of New Ginsenol-Like Compounds with High Antiviral Activity. *Molecules* **2021**, *26*, 6794, doi:10.3390/molecules26226794.

36. de Castro, S.; Ginex, T.; Vanderlinden, E.; Laporte, M.; Stevaert, A.; Cumella, J.; Gago, F.; Camarasa, M.J.; Luque, F.J.; Naesens, L.; et al. N-benzyl 4,4-disubstituted piperidines as a potent class of influenza H1N1 virus inhibitors showing a novel mechanism of hemagglutinin fusion peptide interaction. *Eur. J. Med. Chem.* **2020**, *194*, 112223, doi:10.1016/j.ejmech.2020.112223.

37. Kim, J. Il; Lee, S.; Lee, G.Y.; Park, S.; Bae, J.-Y.; Heo, J.; Kim, H.-Y.; Woo, S.-H.; Lee, H.U.; Ahn, C.A.; et al. Novel Small Molecule Targeting the Hemagglutinin Stalk of Influenza Viruses. *J. Virol.* **2019**, *93*, doi:10.1128/JVI.00878-19.

38. Zhao, X.; Li, R.; Zhou, Y.; Xiao, M.; Ma, C.; Yang, Z.; Zeng, S.; Du, Q.; Yang, C.; Jiang, H.; et al. Discovery of Highly Potent Pinanamine-Based Inhibitors against Amantadine- and Oseltamivir-Resistant Influenza A Viruses. *J. Med. Chem.* **2018**, *61*, 5187–5198, doi:10.1021/acs.jmedchem.8b00042.

39. Adedeji, A.O.; Severson, W.; Jonsson, C.; Singh, K.; Weiss, S.R.; Sarafianos, S.G. Novel Inhibitors of Severe Acute Respiratory Syndrome Coronavirus Entry That Act by Three Distinct Mechanisms. *J. Virol.* **2013**, *87*, 8017–8028, doi:10.1128/JVI.00998-13.

40. Razizadeh, M.; Nikfar, M.; Liu, Y. Small molecule therapeutics to destabilize the ACE2-RBD complex: A molecular dynamics study. *Biophys. J.* **2021**, *120*, 2793–2804, doi:10.1016/j.bpj.2021.06.016.

41. Deganutti, G.; Prischi, F.; Reynolds, C.A. Supervised molecular dynamics for exploring the druggability of the SARS-CoV-2 spike protein. *J. Comput. Aided. Mol. Des.* **2021**, *35*, 195–207, doi:10.1007/s10822-020-00356-4.

42. Aherfi, S.; Pradines, B.; Devaux, C.; Honore, S.; Colson, P.; Scola, B. La; Raoult, D. Drug repurposing against SARS-CoV-1, SARS-CoV-2 and MERS-CoV. *Future Microbiol.* **2021**, *16*, 1341–1370, doi:10.2217/fmb-2021-0019.

43. Banerjee, S.; Yadav, S.; Banerjee, S.; Fakayode, S.O.; Parvathareddy, J.; Reichard, W.; Surendranathan, S.; Mahmud, F.; Whatcott, R.; Thammathong, J.; et al. Drug Repurposing to Identify Nilotinib as a Potential SARS-CoV-2 Main Protease Inhibitor: Insights from a Computational and In Vitro Study. *J. Chem. Inf. Model.* **2021**, *61*, 5469–5483, doi:10.1021/acs.jcim.1c00524.

44. Ruan, Z.; Liu, C.; Guo, Y.; He, Z.; Huang, X.; Jia, X.; Yang, T. SARS‐CoV‐2 and SARS‐CoV: Virtual screening of potential inhibitors targeting RNA‐dependent RNA polymerase activity (NSP12). *J. Med. Virol.* **2021**, *93*, 389–400, doi:10.1002/jmv.26222.

45. Mediouni, S.; Mou, H.; Otsuka, Y.; Jablonski, J.A.; Adcock, R.S.; Batra, L.; Chung, D.-H.; Rood, C.; de Vera, I.M.S.; Rahaim Jr., R.; et al. Identification of potent small molecule inhibitors of SARS-CoV-2 entry. *SLAS Discov.* **2022**, *27*, 8–19, doi:10.1016/j.slasd.2021.10.012.

46. Wang, L.; Wu, Y.; Yao, S.; Ge, H.; Zhu, Y.; Chen, K.; Chen, W. zhang; Zhang, Y.; Zhu, W.; Wang, H. yang; et al. Discovery of potential small molecular SARS-CoV-2 entry blockers targeting the spike protein. *Acta Pharmacol. Sin.* **2021**, doi:10.1038/s41401-021-00735-z.

47. Wei, T. zi; Wang, H.; Wu, X. qing; Lu, Y.; Guan, S. hui; Dong, F. quan; Dong, C. le; Zhu, G. li; Bao, Y. zhou; Zhang, J.; et al. In Silico Screening of Potential Spike Glycoprotein Inhibitors of SARS-CoV-2 with Drug Repurposing Strategy. *Chin. J. Integr. Med.* **2020**, *26*, 663–669, doi:10.1007/s11655-020-3427-6.

48. Kalhor, H.; Sadeghi, S.; Abolhasani, H.; Kalhor, R.; Rahimi, H. Repurposing of the approved small molecule drugs in order to inhibit SARS-CoV-2 S protein and human ACE2 interaction through virtual screening approaches. *J. Biomol. Struct. Dyn.* **2022**, *40*, 1299–1315, doi:10.1080/07391102.2020.1824816.

49. Fantini, J.; Chahinian, H.; Yahi, N. Synergistic antiviral effect of hydroxychloroquine and azithromycin in combination against SARS-CoV-2: What molecular dynamics studies of virus-host interactions reveal. *Int. J. Antimicrob. Agents* **2020**, *56*, 106020, doi:10.1016/j.ijantimicag.2020.106020.

50. Braz, H.L.B.; Silveira, J.A. de M.; Marinho, A.D.; de Moraes, M.E.A.; Moraes Filho, M.O. de; Monteiro, H.S.A.; Jorge, R.J.B. In silico study of azithromycin, chloroquine and hydroxychloroquine and their potential mechanisms of action against SARS-CoV-2 infection. *Int. J. Antimicrob. Agents* **2020**, *56*, 106119, doi:10.1016/j.ijantimicag.2020.106119.

51. Colson, P.; Rolain, J.-M.; Lagier, J.-C.; Brouqui, P.; Raoult, D. Chloroquine and hydroxychloroquine as available weapons to fight COVID-19. *Int. J. Antimicrob. Agents* **2020**, *55*, 105932, doi:10.1016/j.ijantimicag.2020.105932.

52. Fantini, J.; Di Scala, C.; Chahinian, H.; Yahi, N. Structural and molecular modelling studies reveal a new mechanism of action of chloroquine and hydroxychloroquine against SARS-CoV-2 infection. *Int. J. Antimicrob. Agents* **2020**, *55*, 105960, doi:10.1016/j.ijantimicag.2020.105960.

53. Padhi, A.K.; Seal, A.; Khan, J.M.; Ahamed, M.; Tripathi, T. Unraveling the mechanism of arbidol binding and inhibition of SARS-CoV-2: Insights from atomistic simulations. *Eur. J. Pharmacol.* **2021**, *894*, doi:10.1016/j.ejphar.2020.173836.

54. Vankadari, N. Arbidol: A potential antiviral drug for the treatment of SARS-CoV-2 by blocking trimerization of the spike glycoprotein. *Int. J. Antimicrob. Agents* **2020**, *56*, 105998, doi:10.1016/j.ijantimicag.2020.105998.

55. Borisevich, S.S.; Khamitov, E.M.; Gureev, M.A.; Yarovaya, O.I.; Rudometova, N.B.; Zybkina, A. V.; Mordvinova, E.D.; Shcherbakov, D.N.; Maksyutov, R.A.; Salakhutdinov, N.F. Simulation of Molecular Dynamics of SARS-CoV-2 S-Protein in the Presence of Multiple Arbidol Molecules: Interactions and Binding Mode Insights. *Viruses* **2022**, *14*, 119, doi:10.3390/v14010119.

56. Musarrat, F.; Chouljenko, V.; Dahal, A.; Nabi, R.; Chouljenko, T.; Jois, S.D.; Kousoulas, K.G. The anti-HIV drug nelfinavir mesylate (Viracept) is a potent inhibitor of cell fusion caused by the SARSCoV-2 spike (S) glycoprotein warranting further evaluation as an antiviral against COVID-19 infections. *J. Med. Virol.* **2020**, *92*, 2087–2095, doi:10.1002/jmv.25985.

57. Huynh, T.; Wang, H.; Luan, B. In Silico Exploration of the Molecular Mechanism of Clinically Oriented Drugs for Possibly Inhibiting SARS-CoV-2’s Main Protease. *J. Phys. Chem. Lett.* **2020**, *11*, 4413–4420, doi:10.1021/acs.jpclett.0c00994.

58. Ghasemlou, A.; Uskoković, V.; Sefidbakht, Y. Exploration of potential inhibitors for SARS‐CoV‐2 Mpro considering its mutants via structure‐based drug design, molecular docking, MD simulations, MM/PBSA, and DFT calculations. *Biotechnol. Appl. Biochem.* **2022**, doi:10.1002/bab.2369.

59. Li, H.; Cheng, C.; Shi, S.; Wu, Y.; Gao, Y.; Liu, Z.; Liu, M.; Li, Z.; Huo, L.; Pan, X.; et al. Identification, optimization, and biological evaluation of 3-O-β-chacotriosyl ursolic acid derivatives as novel SARS-CoV-2 entry inhibitors by targeting the prefusion state of spike protein. *Eur. J. Med. Chem.* **2022**, *238*, 114426, doi:10.1016/j.ejmech.2022.114426.

60. Yarovaya, O.I.; Shcherbakov, D.N.; Borisevich, S.S.; Sokolova, A.S.; Gureev, M.A.; Khamitov, E.M.; Rudometova, N.B.; Zybkina, A. V.; Mordvinova, E.D.; Zaykovskaya, A. V.; et al. Borneol Ester Derivatives as Entry Inhibitors of a Wide Spectrum of SARS-CoV-2 Viruses. *Viruses* **2022**, *14*, 1295, doi:10.3390/v14061295.
